# AlphaFold2 SLiM screen for LC3-LIR interactions in autophagy

**DOI:** 10.1080/15548627.2025.2493999

**Published:** 2025-05-04

**Authors:** Jan Felix Maximilian Stuke, Gerhard Hummer

**Affiliations:** a Department of Theoretical Biophysics, Max Planck Institute of Biophysics, Frankfurt am Main, Germany; b Institute of Biophysics, Goethe University Frankfurt, Frankfurt am Main, Germany

**Keywords:** AIM, Atg8, phosphorylation, prediction, selective autophagy receptor, SUMO-SIM interaction

## Abstract

In selective macroautophagy/autophagy, cargo recruitment is mediated by MAP1LC3/LC3-interacting regions (LIRs)/Atg8-family interacting motifs (AIMs) in the cargo or cargo receptor proteins. The binding of these motifs to LC3/Atg8 proteins at the phagophore membrane is often modulated by post-translational modifications, especially phosphorylation. As a challenge for computational LIR predictions, sequences may contain the short canonical (W/F/Y)XX(L/I/V) motif without being functional. Conversely, LIRs may be formed by non-canonical but functional sequence motifs. AlphaFold2 has proven to be useful for LIR predictions, even if some LIRs are missed and proteins with thousands of residues reach the limits of computational feasibility. We present a fragment-based approach to address these limitations. We find that fragment length and phosphomimetic mutations modulate the interactions predicted by AlphaFold2. Systematic fragment screening for a range of target proteins yields structural models for interactions that AlphaFold2 and AlphaFold3 fail to predict for full-length targets. We provide guidance on fragment choice, sequence tuning, LC3 isoform effects, and scoring for optimal LIR screens. Finally, we also test the transferability of this general framework to SUMO-SIM interactions, another type of protein-protein interaction involving short linear motifs (SLiMs).

**Abbreviations**: 2-HP-LIR: ncLIR binding either or both HPs with non-canonical residues; AIM: Atg8-family interacting motif; ap. LIR: antiparallel LIR; *A.t*.; *Arabidopsis thaliana*; AT5G06830/C53 (*A.t*.): CDK5RAP3-like protein; Atg8/ATG8: autophagy related 8, in yeast and plants, respectively; ATG8CL: ATG8C-like of *Solanum tuberosum* (potato); ATG8E: ATG8e of *A.t*.; Av. num. of contacts: average number of heavy atom contacts; BCL2: BCL2 apoptosis regulator; BNIP3: BCL2 interacting protein 3; CALCOCO2/NDP52: calcium binding and coiled-coil domain 2; CALR: calreticulin; can. LIR: canonical LIR; CDF: cumulative distribution function; CDK5RAP3/C53 (*H.s*.): CDK5 regulatory subunit associated protein 3; [DE]W[DE]-LIR: TRIM5-like ncLIR; DSK2A: ubiquitin domain-containing protein DSK2a; FUNDC1: FUN14 domain containing 1; GABARAP: GABA type A receptor-associated protein; HP0/1/2: hydrophobic pocket 0/1/2; HP0-LIR: ncLIR engaging HP0; *H.s*.; *Homo sapiens*; lcLIR: low-confidence LIR (ncLIR not similar to previously characterized ncLIRs); LDS: LIR-docking site; LIR: LC3-interacting region; LO score: length-weighted fraction of occurrence score; MAP1LC3/LC3: microtubule associated protein 1 light chain 3; MAP1LC3B/LC3B: microtubule associated protein 1 light chain 3 beta; MD: molecular dynamics; MEFV/pyrin: MEFV innate immunity regulator, pyrin; minPAE: minimum PAE; MSA: multiple sequence alignment; ncLIR: non-canonical LIR; NPC: nuclear pore complex; Nup159: nucleoporin 159; NUP214: nucleoporin 214; OPTN: optineurin; other@LDS: other interaction proximal to the LIR-docking site; PAE: predicted aligned error; PDCD6IP: programmed cell death 6 interacting protein; PDF: probability distribution function; pLDDT: predicted local-distance difference test; PLEKHM1: pleckstrin homology and RUN domain containing M1; PTM: post-translational modification; sAIM: shuffled AIM (ncLIR with shuffled motif); seq.: sequence; SIM: SUMO-interacting motif; SLiM: short linear motif; SMN1/SMN: survival of motor neuron 1, telomeric; ST: phosphomimetic; STBD1: starch binding domain 1; STK3: serine/threonine kinase 3; SUMO: small ubiquitin like modifier; TBC1D2/TBC1D2A: TBC1 domain family member 2; TEX264: testis expressed 264, ER-phagy receptor; TRIM5/TRIM5α: tripartite motif-containing protein 5; UDS: UIM-docking site; UIM: ubiquitin-interacting motif; UIMC1/RAP80: ubiquitin interaction motif containing 1; ULK1: unc-51 like autophagy activating kinase 1; ULK2: unc-51 like autophagy activating kinase 2; WT: wild type

## Introduction

Selective macroautophagy/autophagy is central to homeostasis in eukaryotic cells. Autophagy is used to rid cells of pathogens [1], recycle organelles [2,3], and degrade protein condensates [4] and aggregates [5]. The cargo is enveloped by a double membrane, the phagophore, which closes and matures to form the autophagosome. Subsequently, the autophagosome fuses with a lysosome (or the vacuole in fungi and plants), leading to the degradation of the cargo [6,7]. Selective autophagy is associated with several pathophysiological processes, including neurodegenerative diseases and cancer [8].

ATG8 (autophagy related 8) proteins – MAP1LC3/LC3 (microtubule associated protein 1 light chain 3) and GABARAP (GABA type A receptor-associated protein) families in mammals, Atg8 in yeast, ATG8 in plants – facilitate the recruitment of cargo to the phagophore by binding to LC3-interacting regions (LIRs)/Atg8-family interacting motifs (AIMs) [7,9]. In the following, we will refer to LC3 and LIR as generic names for these proteins and motifs, respectively. LC3 proteins are small proteins with a core fold similar to ubiquitin. They are modified via lipidation and anchored to the phagophore membrane [10]. They bind either directly to cargo proteins or to cargo receptor proteins, which bind to both LC3 and cargo proteins. Segments of cargo proteins binding directly to LC3s are called intrinsic receptors. Nup159 (nucleoporin 159), for instance, is a part of the nuclear pore complex (NPC) that has been shown to interact directly with Atg8 and mediate selective autophagy of the NPC [11]. The regions in (intrinsic) cargo receptors that bind to LC3s are – typically though not exclusively – LIRs. As an alternative, proteins with ubiquitin-interacting motif (UIM)-like sequences can bind to LC3’s UIM-docking site (UDS) [12,13].

Canonical LIRs are short linear sequences that bind to two hydrophobic pockets in LC3. The canonical LIR motif consists of four residues that follow the pattern Θ-X-X-Γ. Θ (=W, F or Y) binds in hydrophobic pocket 1 (HP1) and Γ (=L, I or V) in hydrophobic pocket 2 (HP2). Additionally, the LIR motif forms a parallel β-sheet with the β2-strand of the LC3 protein [13]. Besides canonical LIRs, non-canonical LIRs (ncLIRs) have been identified that can deviate from this interaction mode in various ways [14]; e.g., they have non-canonical residues engaging the hydrophobic pockets [15], have a “shuffled” motif [16], bind only one of the hydrophobic pockets, e.g., the CLIR motif of CALCOCO2/NDP52 (calcium binding and coiled-coil domain 2) or STK3 (serine/threonine kinase 3) [17,18], engage a third hydrophobic pocket (termed HP0) [19], and/or have an α-helical structure, e.g., in TRIM5/TRIM5α (tripartite motif-containing protein 5) and BCL2 (BCL2 apoptosis regulator) [20,21]. Furthermore, there are also non-continuous LIRs, in which the LC3 binding residues are distributed over a wide range of the protein sequence [22]. The possibility of an antiparallel Γ-X-X-Θ LIR motif has also been demonstrated with an artificial peptide [23]. Most LIRs reside within intrinsically disordered regions, allowing for conformational flexibility when adapting to the LIR-docking site (LDS), and, hence, promiscuity of LC3 proteins [24]. Canonical and non-canonical core LIR motifs are often flanked by acidic and/or phosphorylatable residues [13].

Phosphorylation modulates LC3-LIR interactions. For multiple LIRs it has been shown that their binding to LC3s can be influenced by phosphorylation of residues in close proximity to the LIR [25,26]. Structural and binding affinity studies suggest that these phosphorylated residues interact with a range of positively charged residues in the LC3 protein and thereby stabilize the interaction. Still, in some cases phosphorylation has a negative regulatory effect [3].

A variety of experimental methods can be deployed to identify and characterize functional LIR motifs. RNAi-based screenings, yeast two-hybrid assays, and mass spectrometry-based proteomics are examples of tools used to identify proteins interacting with LC3s. Mutating suspected LIRs and observing the effect on binding can serve as way to identify the exact binding site(s) [8]. To show functionality in the context of selective autophagy, these motifs can be tested via *in vivo* mutation studies [11,27]. For biophysical characterizations, e.g., by calorimetry, and structural studies, usually not the full-length protein is used. Rather, these studies are conducted with short peptide fragments containing the respective LIRs [25,26,28]. Computational methods complement these experimental approaches by predicting LIRs and thereby limiting the search space.

AlphaFold2 [29] has emerged as a powerful new computational tool to aid in the prediction of LIRs and to focus experimental tests, complementing sequence-based computational methods for the prediction of LIRs, e.g., iLIR [30]. As possible challenges in LIR prediction, the sequences may contain the canonical motif without being functional or be functional with non-canonical motifs. Ibrahim et al. [31] found AlphaFold2 Multimer [32] to perform well in predicting LC3-LIR interactions, albeit with room for improvement. Some interactions were not detected, multiple LIRs in one protein required workarounds, and post-translational modifications (PTMs) were not accounted for. Alternatively, a recent data-driven approach has made use of AlphaFold2 as one part of a pipeline to identify novel selective autophagy receptors [33]. Coming from a different angle, various new approaches have been developed to make use of AlphaFold2 Multimer’s ability in the prediction of interactions between proteins and peptides or protein fragments [34,35].

Besides LC3-LIR interactions, there are other protein-protein interactions involving short linear motifs (SLiMs) that might be targetable with similar computational approaches. Small ubiquitin-like modulators (SUMOs) are small proteins with a ubiquitin-like core fold and a short disordered N-terminal region [36]. They are linked to substrate proteins via an isopeptide bond as a PTM called SUMOylation. Proteins with SUMO interacting motifs (SIMs) can bind to SUMO’s SIM binding site [37,38]. SUMOylation has been shown to modulate protein complex and condensate formation in multiple cellular processes [39]. Hence, the prediction of SIMs is of high interest as well, and has been the target of previous and recent method developments [40–42].

Here, we present a fragment-based AlphaFold2 screen to find novel LIR candidates via the prediction of LC3-LIR complexes. We scan fragments of the target protein with varying lengths for interactions with LC3 proteins. Furthermore, we also attempt to capture the effect of phosphorylations by introducing phosphomimetic mutations. We show that both fragment length and phosphomimetic mutations modulate the models created by AlphaFold2. Systematic fragment screening for a range of target proteins yields structural models for interactions that AlphaFold2 fails to predict for full-length targets. Finally, we also test the transferability of this framework by applying it to SUMO-SIM interactions.

## Results

### Target fragmentation improves AlphaFold2 predictions of LC3-LIR complexes

To highlight the power but also limitations of AlphaFold for the prediction of LC3-LIR interactions from full-length sequences, we performed full-length predictions for four different systems. A yeast system (Atg8 with Nup159), a plant system (ATG8CL [ATG8C-like of *Solanum tuberosum*] with Joka2), and two mammalian systems (MAP1LC3B/LC3B [microtubule associated protein 1 light chain 3 beta] with OPTN [optineurin] and GABARAP with CALR [calreticulin]). Three of the these systems have also been tested in a previous sophisticated study of AlphaFold2’s ability to predict LC3-LIR interactions from full-length sequences [31]. In addition to AlphaFold2 (versions 2.2 and 2.3), we used the recently developed AlphaFold3 [43] for full-length predictions, if predictions with AlphaFold2 were unsuccessful.

**Figure 1.**
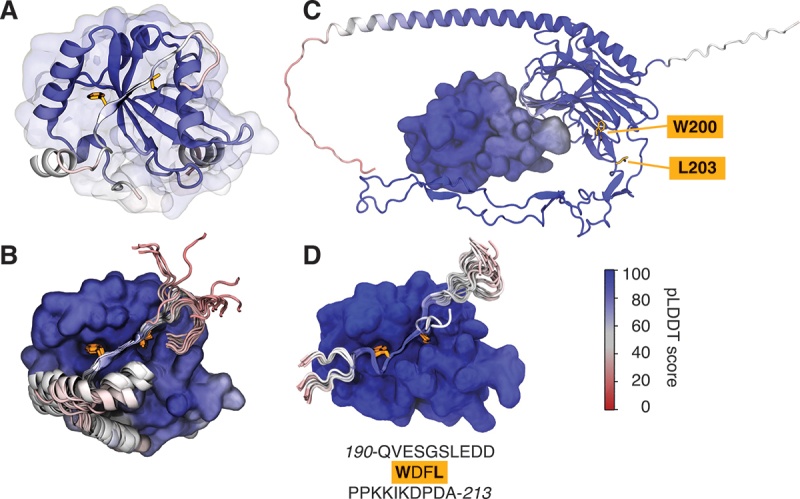
AlphaFold2 multimer predictions with fragments can capture LC3-LIR interactions that are not found when using full-length sequences. (A, B) LC3-OPTN interaction is captured by AlphaFold2.3. (A) The highest scoring (“top”) model of an AlphaFold2.3 prediction of the interaction between LC3B (cartoon with transparent surface representation) and full-length OPTN (cartoon representation, with LIR residues F178 and I181 highlighted in orange licorice). Only the region around the LIR – residues 164–190 – is shown for clarity. (B) All 25 models of the prediction are shown, aligned on LC3B (here in surface representation). Only LC3B from the top model is shown for clarity. (C) AlphaFold2.3 does not capture the interaction of GABARAP with full-length CALR. Shown is the top model of an AlphaFold2.3 prediction of the interaction between GABARAP (surface representation) and full-length CALR (cartoon representation, with LIR residues W200 and L203 highlighted in orange licorice). (D) AlphaFold2.3 captures interactions between GABARAP and a LIR-containing fragment of CALR. Shown are 25 models of an AlphaFold2.3 prediction of the interaction between GABARAP (surface representation) and a 24-amino-acid CALR fragment containing the canonical LIR (cartoon representation, with LIR residues W200 and L203 highlighted in orange licorice), aligned on GABARAP. Only GABARAP from the top model is shown for clarity. All structures are colored by predicted local-distance difference test (pLDDT) score.

We found that AlphaFold2 Multimer [32] readily predicts some known LC3-LIR complex structures when using full-length sequences, but not all, consistent with earlier reports [31]. The interaction of the OPTN LIR_178*−*181_ with LC3B [25] was captured with high confidence (Figure 1A,B). As evidence for advances in data and software, AlphaFold2.3 also predicted a complex between the experimentally confirmed AIM1_1078*−*1081_ of Nup159 and Atg8 [11,28] (Figure S1A), which was not yet captured by AlphaFold2.2.

Following this trend, AlphaFold3 consistently predicted an interaction between Joka2’s experimentally confirmed LIR [44] and ATG8CL, but placed the Θ and Γ residues of the motif only close to yet not in HP1 and HP2, respectively (Figure S1B). This interaction had not been found by AlphaFold2.2 and 2.3, again consistent with previous reports [31]. By contrast, the validated interaction of CALR with GABARAP [45,46] was captured neither by AlphaFold2.2 and 2.3 (Figure 1C and S1C, D), as found before [31], nor by AlphaFold3 (Figure S1E). So far, we had used full-length protein sequences in all predictions.

By shortening the amino-acid sequence containing a putative LIR into a fragment, we expanded the set of LC3-LIR complexes captured by AlphaFold2, as we will show for one example. Experimentally, LIRs are often probed in the form of short peptide fragments, especially in structural studies [25,26]. Therefore, we reasoned that AlphaFold2 might perform well on short peptide fragments. Indeed, it was able to predict the experimentally validated LIR in an LC3-LIR interaction for GABARAP and a 24-residue CALR fragment (Figure 1D).

**Figure 2.**
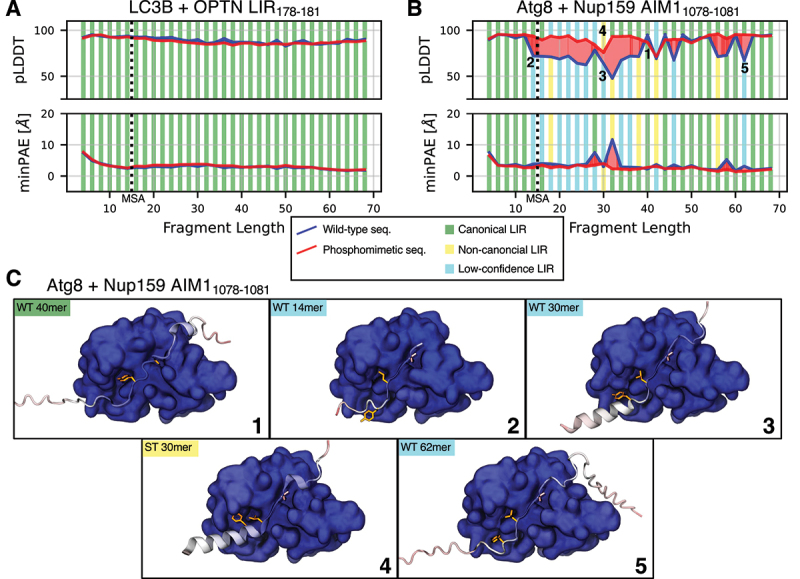
Fragment length and phosphomimetic mutations modulate the prediction of LC3-LIR interactions with AlphaFold2. Predicted local-distance difference test (pLDDT) score, minimum predicted aligned error (minPAE), and binding mode for the four core LIR/AIM residues in AlphaFold2.3 predictions of (A) LC3B and OPTN fragments centered on the LIR_178*−*181_ and (B) Atg8 and Nup159 fragments centered on the AIM1_1078*−*1081_. Fragments were varied (i) in length (x-axis) and (ii) by the introduction of phosphomimetic S/T to E mutations for experimentally confirmed phosphosites (red line) compared to wild type (WT, blue line). The vertical dotted lines indicate the minimum length required for the MSA of AlphaFold2. Stripes above the pLDDT curve/below the minPAE curve indicate the binding mode of the respective fragment with phosphomimetic mutations. Vertical bars below the pLDDT curve/above the minPAE curve indicate the binding mode of the respective WT fragment (green: canonical LIR; yellow: ncLIR; light blue: lcLIR). (C) structures for some selected fragments from B showing one canonical, one non-canonical, and three low-confidence LC3-LIR interactions. Nup159 fragments are shown in cartoon representation with Y1078 and L1081 highlighted in orange, and I1084 in pink licorice. Atg8 is shown in surface representation. All structures are colored by pLDDT score. Numbers in B correspond to structures in C.

Varying the fragment length modulated the structural predictions of AlphaFold2, but the strength of the effect was system dependent. To demonstrate this, we systematically varied the fragment length for the LC3B-OPTN and Atg8-Nup159 systems (Figure 2). We centered the fragments on the experimentally confirmed LIR_178*−*181_ and AIM1_1078*−*1081_ for OPTN and Nup159, respectively. The length varied from 4—just the core LIR motif – to 68 residues. We compared AlphaFold’s quality scores, predicted local-distance difference test (pLDDT) score and minimum predicted aligned error (minPAE), of the core LIR motif for every generated fragment. The shortest segments (*<*15 residues), for which AlphaFold2 fails to generate an MSA, tended to form high-scoring canonical LC3-LIR complexes in both systems. For short to medium length fragments (approximately 15 to 40 residues), there was a weak decline in scoring for OPTN, and a strong decline for Nup159, before scores partially recovered for long (*>*40 residues) fragments (Figure 2A and B, blue lines). For Nup159 the fragment length also modulated whether a canonical interaction, a non-canonical interaction similar to previously described ones, or a so far not described “low-confidence” (lcLIR) interaction was predicted (Figure 2B, C). Here, while in the non-canonical interaction mode HP1 was engaged by a non-canonical residue, in the low-confidence mode interactions at HP1 were only superficial. Since the motif lacked other compensating features found in LIR structures only binding HP2, e.g., the additional hydrophobic residues of the CLIR [17], we classified it as a low-confidence non-canonical interaction.

### Phosphomimetic mutations improve AlphaFold2 predictions of LC3-LIR complexes

Phosphorylation is a modulator of LC3-LIR interactions, with experimentally resolved structures of LC3-LIR complexes frequently containing phosphomimetic mutations or phosphorylated residues flanking the LIR [25,26]. We therefore tested if the introduction of phosphomimetic mutations – AlphaFold2 cannot handle phosphorylated residues, so we could not test those – influences AlphaFold2’s prediction behavior for LC3-LIR complex structures in a way that would be useful in screening for potential LIRs. We introduced phosphomimetic S/T to E mutations for all S and T residues in our fragments listed in dbPTM [47], a database for PTMs. For OPTN LIR_178*−*181_ the effect on the already high scores was negligible (Figure 2A, red lines), for Nup159 AIM1_1078*−*1081_ scores tended to improve, in some cases greatly, and seven more fragments captured the canonical interfaces (Figure 2B, red lines). The effect was similar when using AlphaFold2.2 instead of 2.3, albeit with mostly lower model quality (Figure S2A, B). Overall, phosphomimetic mutations emerged as a tool to modulate an AlphaFold2 screen and identify otherwise missed LC3-LIR interactions.

### Systematic screening over multiple targets reveals known LIRs and novel LIR candidates

We aimed to exploit the observed effects of fragment length and phosphomimetic mutations to design a systematic screen for the identification of LIR motifs (Figure S2C). From the sequence of a given target protein, we generated fragments of a defined length with 75% overlap between them. We then repeated the process with a sequence of the protein in which all S and T residues expected to be phosphorylated were mutated to E. For all resulting unique fragments, structural complexes were predicted with AlphaFold2 Multimer. Every resulting complex structure was assessed based on AlphaFold2’s quality scores and the type of interaction (see section *Evaluation of generated structures* in the methods for details), similar to the fragment-length screen. To test if the predicted LIR is surface-accessible to bind to LC3, we used the AlphaFold2 predicted structure of the full-length target protein in isolation. As metrics for accessibility we calculated residue depth (r.d.) [48,49] and secondary structure, where both were only assigned to residues with pLDDT scores above 70 in the full-length structure (irrespective of the scores in the fragments).

We performed this screen on LC3B and OPTN, GABARAP and CALR, and Atg8 and Nup159. For each of these systems we performed runs with short, medium, and long fragments (16, 32, and 52 residues, respectively). We did not include very short sequences, for which no MSA can be calculated, to minimize the risk for false positives. The screen found the experimentally confirmed LIRs in all three systems. Interactions between fragments and LC3s were favored for shorter fragments (Figure S3A, B, C), suggesting a potential trade-off between specificity and sensitivity.

**Figure 3.**
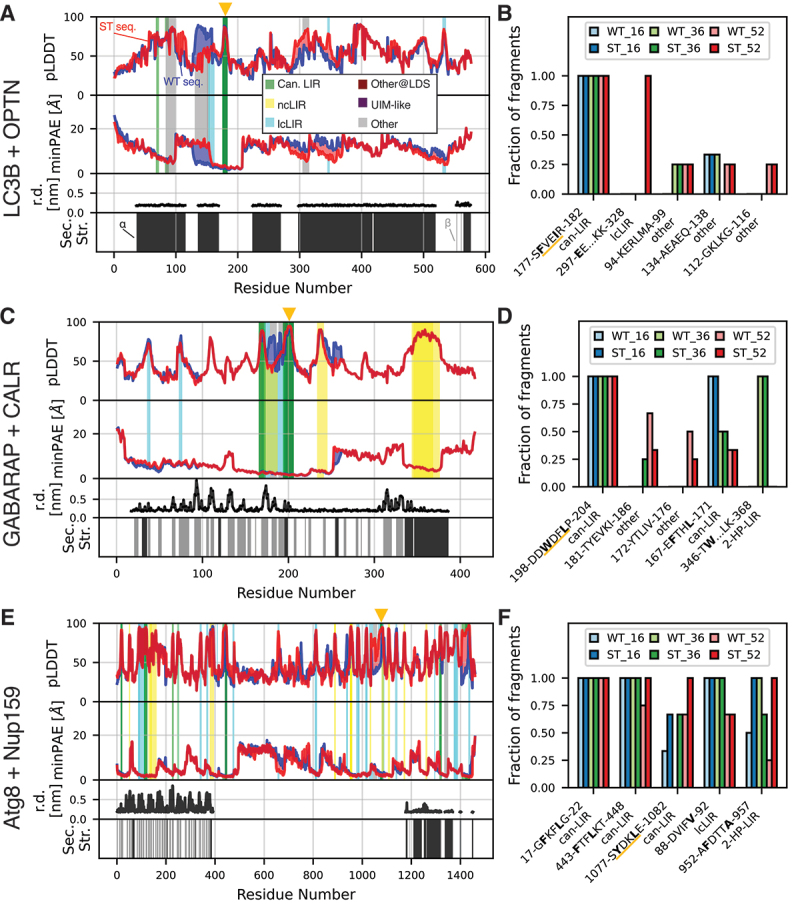
Scanning over the entire sequence of target proteins with fragment-LC3 AlphaFold2 multimer predictions reveals known LIRs and novel candidate LIRs. (A) systematic screen of the interaction between 36-residue fragments of OPTN with LC3B. The fragments have a 75% overlap. We performed the screen for the WT sequence, and an ST sequence, in which S/T to E mutations for experimentally confirmed phosphosites were introduced. Predicted local-distance difference test (pLDDT) score, minimum predicted aligned error (minPAE), and binding mode for the interacting residues are shown (minimum minPAE from all fragments covering each residue and its respective pLDDT score). Stripes above the pLDDT curve/below the minPAE curve indicate the binding mode of the respective fragment with phosphomimetic mutations. Stripes below the pLDDT curve/above the minPAE curve indicate the binding mode of the respective WT fragment. Additionally, we report the residue depth (r.d.) and secondary structure (sec. str.) if the pLDDT score of the respective residue in the full-length structure is ≥ 70, as calculated from the AlphaFold2 predicted structure for the full-length protein to add structural context to the sequence (black: α-helix; gray: β-sheet). The experimentally confirmed LIRs are indicated at the top by orange triangles. (B) summary of predicted LIRs from the screen in A and two additional screens using the same setup, but 16-residue and 52-residue fragments, respectively. Shown are the top five predicted LIRs, sorted by relative occurrence in longer fragments (52mers *>* 36mers *>* 16mers). Residues binding in hydrophobic pocket 0, 1 and 2 are highlighted in bold. The interaction type is indicated below each motif. The experimentally confirmed LIR is underlined in orange. (C and D) results of an analogous screen for CALR and GABARAP. (E and F) results of an analogous screen for Nup159 and Atg8.

We combined the information from the three screens performed for LC3B and OPTN (Figure 3A and S3A) and grouped similar interactions (Figure 3B). The validated LIR_178*−*181_ was consistently predicted in all fragments that contain it, for all fragment lengths, and for WT and phosphomimetic sequences. Furthermore, a low residue depth and no secondary structure for the motif in the full-length protein suggested that it is not buried deep in a folded domain. Similarly, the validated CALR LIR_200*−*203_ was found in all fragments containing it (Figure 3C,D and S3B), and lies at the edge of a folded region, suggesting that it is partially hidden (Figure 3C, compare to the structure shown in Figure 1C and S1C). Nup159 AIM1_1078*−*1081_ was also reliably predicted, though not in all fragments containing it (Figure 3E,F and S3C). For this LIR, phosphomimetic mutations substantially increased the predictive power. The fraction of fragments for which the LIR was predicted rose from 0.33 to 0.67 for 16mers, from 0.0 to 0.67 for 36mers, and from 0.67 to 1.0 for 52mers. AIM1 is located in an unstructured segment of the protein (Figure 3E). Contrary to OPTN and CALR, the experimentally confirmed LIR was not the most reliably predicted one in Nup159.

Instead, the screen suggested novel LIR candidates for Nup159 and other systems. For OPTN these alternative interacting motifs occurred only in a small fraction of the fragments and lie within α-helical parts of the protein, leaving the evidence rather weak. For CALR, a second LIR was reliably predicted, though it is located in a folded region and, hence, likely not available as long as CALR is folded. By contrast, for Nup159 the screen predicted a canonical LIR (residues 443–448; also predicted by iLIR [30]) and a ncLIR (952–956), respectively, with high reliability and in unstructured regions of the protein. Two more LIRs predicted with high consistency are located within or at the edge of the folded N-terminal domain of Nup159. Nup159 also contains four LIR motifs (AIM2, AIM3, AIM4, and AIM5) that were previously predicted but, in experiment, found to be nonfunctional [11]. Hence, they serve as an important negative control. Our screen found none of them in the top 5, and only two of them in the top 15 (Figure S3D). Instead, our screen was dominated by the experimentally confirmed AIM1.

We aimed to find a single scoring metric, summarizing the results for multiple fragment lengths, to facilitate the interpretation of the screen results. For this, we created a benchmark set containing the systems tested so far (LC3B and OPTN, GABARAP and CALR, Atg8 and Nup159), as well as nine more systems, which had been studied previously in experiments: GABARAP and STBD1 (starch binding domain 1) [50], GABARAP and BNIP3 (BCL2 interacting protein 3) [51,52], GABARAP and ULK2 (unc-51 like autophagy activating kinase 2) [53], ATG8CL and Joka2 [44], ATG8E and DSK2A (ubiquitin domain-containing protein DSK2a) [54], GABARAP and MEFV/pyrin (MEFV innate immunity regulator, pyrin) [55], GABARAP and TBC1D2/TBC1D2A (TBC1 domain family member 2) [56], ATG8E and AT5G06830/C53 (CDK5RAP3-like protein, *Arabidopsis thaliana*), and GABARAP and *Homo sapiens* (*H.s*.) CDK5RAP3/C53 (CDK5 regulatory subunit associated protein 3) [16]. This set consists of both relatively simple targets containing one confirmed functional canonical LIR, as well as very challenging ones containing multiple confirmed (non-)functional (non-)canonical LIRs. In total, the set contains 22 confirmed functional LIRs (of which 12 are canonical) and 8 confirmed nonfunctional LIRs (all canonical).

**Figure 4.**
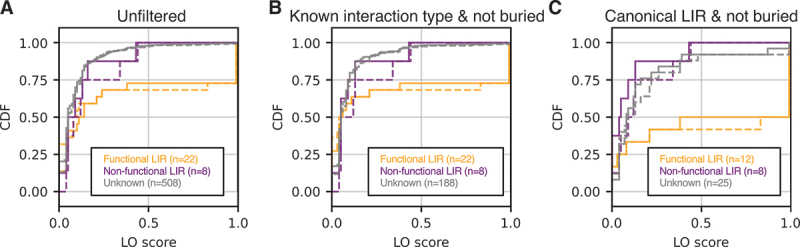
The length-weighted fraction of occurrence (LO) score is a measure of prediction consistency that can discriminate between functional and nonfunctional LIRs. (A) Cumulative distribution function of the LO scores for all predicted and expected motifs in our benchmark set (detailed data shown in Figure 3, Figure 5 and Figure S4) grouped by experimentally confirmed functional motifs (in orange), experimentally confirmed nonfunctional motifs (purple), and motifs with unknown functionality (gray). Solid lines show the scores for WT and dotted lines the scores for phosphomimetic (ST) predictions. Experimentally confirmed or non-confirmed motifs that were not found in the predictions were scored with 0. (B) Same distributions as in A after the predicted motifs have been filtered to only contain non-buried motifs (average residue depth ≤ 0.3 nm) with known interaction modes (canonical LIRs, ncLirs, UIMs). (C) Same distributions as in A after the predicted motifs have been filtered to only contain non-buried canonical LIRs, and the expected motifs to only contain canonical LIRs (expected motifs were not evaluated for whether they are buried or not).

In total, 19 of 22 functional and all 8 nonfunctional LIRs (or a motif overlapping with them) were detected in at least one fragment, as well as 508 potential candidates. While functional LIRs did not have better AlphaFold2.3 scores than nonfunctional ones when comparing the best-scoring fragments for each motif (Figure S4A, B), they were predicted more consistently, as quantified by the length-weighted fraction of occurrence (LO) score (Figure 4A). Notably, no nonfunctional LIR scored higher than 0.5. In an attempt to reduce the number of false positives, we filtered the dataset by only considering accessible (meaning not or only partially buried) motifs (average residue depth ≤ 0.3 nm; for reference, the average r.d. of CALR’s partially buried LIR was 0.24 nm) with known interaction modes (canonical LIR, previously described ncLIRs, UIMs). This led to the loss of 3 functional LIRs but reduced the pool of candidates to 188 (Figure 4B). To give an example, filtering removed the candidate LIR_637*−*640_ in Joka2, which is predicted to be buried in a folded domain (Figure S5A). Since our benchmark set only contains canonical nonfunctional LIRs, we also compared scoring for only canonical LIRs (Figure 4C). Here, scores for functional motifs were strongly increased compared to nonfunctional motifs. This also indicates that functional non-canonical motifs tend to score lower than canonical ones. Interestingly, a small fraction of the candidate LIRs in the group of unknown functionality scored highly, and thus appear to be promising candidates for functional LIRs. Phosphomimetic mutations had a mixed effect on scores for unfiltered sets and sets containing ncLIRs but tended to increase scores for canonical motifs (Figure 4). With only very few exceptions, the vast majority of impactful phosphomimetic mutations – in the sense of changing binding mode or moving the fragment over/under the detection (pLDDT and minPAE) threshold – resided in close proximity to the interacting motif (Figure S4C).

**Figure 5.**
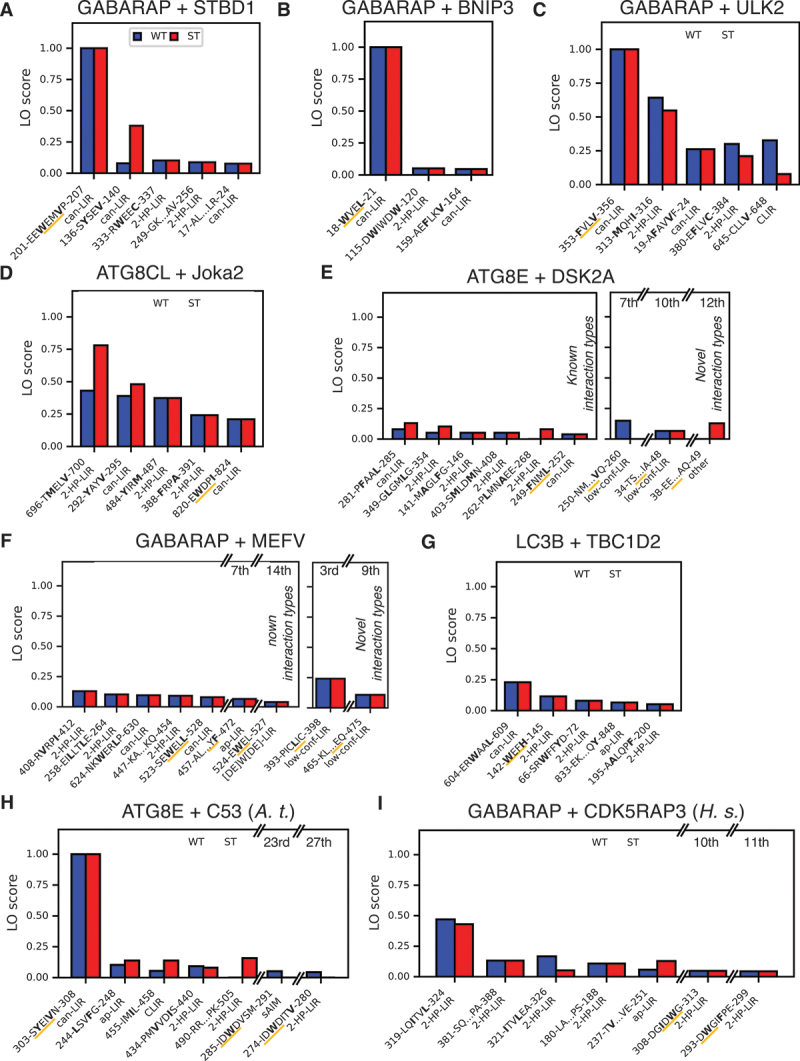
The prediction of LC3-LIR interactions produces mixed results for challenging targets. Results of fragment-prediction screens for (A) GABARAP and STBD1, (B) GABARAP and BNIP3, (C) GABARAP and ULK2, (D) ATG8CL and Joka2, (E) ATG8E and DSK2A, (F) GABARAP and MEFV, (G) LC3B and TBC1D2, (H) ATG8E and C53 (*A. t*.), and (I) GABARAP and CDK5RAP3 (*H.S*.). Shown are the top five (BNIP3 only has three) predicted LIRs with known interaction type (can. LIR, ncLIR, UIM) and an average residue depth ≤ 0.3 nm and all lower-ranking predicted LIRs (rank indicated above respective bars) corresponding to experimentally confirmed functional ones, sorted by LO score (averaged over WT and ST runs). Confirmed functional motifs in novel interaction modes (lcLIR,other) are also shown with the respective rank indicating their position within the group of low-confidence motifs. Residues binding in hydrophobic pocket 0, 1, and 2 are highlighted in bold. The interaction type is indicated below each motif. The experimentally confirmed functional LIRs are underlined in orange.

Following is a detailed look at the different systems in our benchmark set to assess the performance in each individual case. Running the screen with GABARAP against STBD1 (Figure 5A), BNIP3 (Figure 5B), and ULK2 (Figure 5C) captured the experimentally confirmed [50–53]—but structurally unresolved – LIR, respectively, in all fragments. For ATG8CL with Joka2 and ATG8E with DSK2A, the results were less clear. For Joka2, the experimentally confirmed LIR [44] was predicted less consistently, with a couple of other sequences ranking clearly above it (Figure 5D). In DSK2A, we found all three confirmed LIRs [54] (LIR_43*−*46_, LIR_249*−*252_, and LIR_256–259_) in at least one fragment (Figure 5E), with LIR_43*−*46_ occurring in three different binding modes (Figure S5C), LIR_249*−*252_ being ranked sixth, and LIR_256–259_ only occurring in a low-confidence interaction. Interestingly, in the canonical interaction, which contains residues 42–48 as interacting residues, LIR_43*−*46_ had an average residue depth higher than the cutoff of 0.3 nm. The two other interaction modes were longer and contained more accessible residues, lowering the average residue depth below the cutoff. A look at the full-length structure revealed that LIR_43*−*46_ was predicted to be part of the core of a small, folded domain (Figure S5B).

The benchmark set for the screen also contains four very challenging targets containing multiple (non-)canonical LIRs: GABARAP and MEFV [55], LC3B and TBC1D2 [56], ATG8E and C53 (*A.t*.), and GABARAP and CDK5RAP3 (*H.s*.) [16]. For MEFV, our screen picked up the canonical LIR and the two ncLIRs (one of them in two different binding modes; Figure S5D), but these interactions were not reliably predicted (Figure 5F). For TBC1D2, we found one of the two ncLIRs, again with a rather weak signal, but ranking second (Figure 5G). For C53 (*A.t*.) the canonical LIR was predicted consistently, but of the three closely grouped (Figure S5E) ncLIRs only two were predicted in at least one fragment, and none of them ranked in the top 15 (Figure 5H). Like for Nup159, this system contains four nonfunctional LIRs that can be used as negative controls. Three of these were within our top 15 candidates (one in 2 binding modes), being predicted less consistently than the experimentally confirmed canonical LIR (Figure S5F). For CDK5RAP3 (*H.s*.), we found two of the three confirmed ncLIRs, with one of them making it into the top 15 (Figure 5I), but again with low consistency. Interestingly, the only LIR predicted with high consistency (in two different binding modes with parallel or antiparallel 2-HP-LIR, respectively) lies in close proximity to the experimentally identified ones. Both experimental LIRs and the predicted novel candidate LIR_320*−*324_ are located in an unstructured part of the protein, while many of the other signals occurred in domains expected to be folded, even if they were below the r.d. cutoff (Figure S5E).

### Phosphomimetic mutations mimic phosphorylation in molecular dynamics simulations

**Figure 6.**
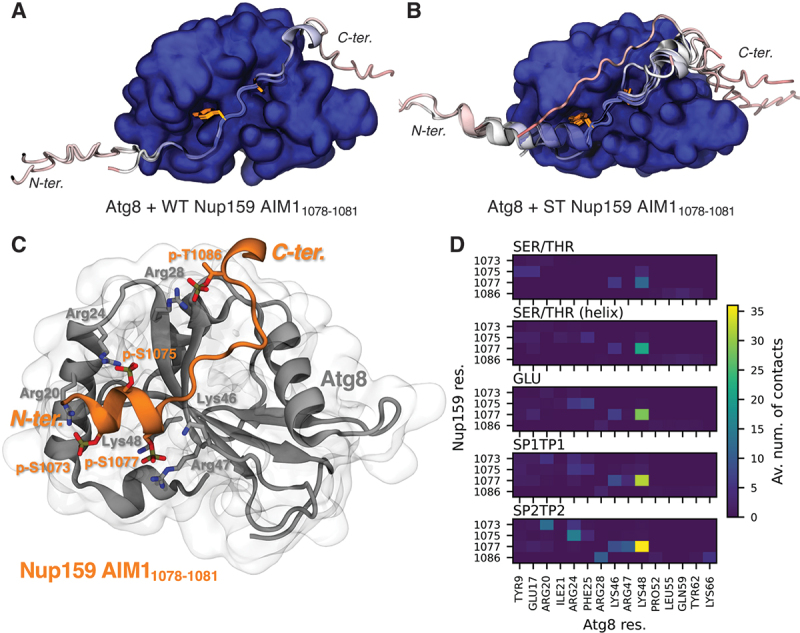
Phosphomimetic mutations alter the structure of AIM1 in Nup159, its interactions with Atg8, and behave similar to real phosphorylations. (A) Top models of all WT Nup159 fragments (cartoon representation) containing AIM1_1078*−*1081_ (Y1078 and L1081 highlighted as orange licorice) and interacting with Atg8 (surface representation) in a canonical LC3-LIR interaction. (B) Top models of all Nup159 fragments with phosphomimetic mutations (cartoon representation) containing AIM1_1078*−*1081_ (Y1078 and L1081 highlighted as orange licorice) and interacting with Atg8 (surface representation) in a canonical LC3-LIR interaction. In A and B only one Atg8 structure is shown for clarity, structures are aligned on Atg8, and colored by predicted local-distance difference test (pLDDT) score. (C) Snapshot structure of Atg8 and a Nup159 AIM1 fragment (both shown in cartoon representation) with four phosphorylations after 450 ns of MD simulation, showing some of the most prominent charge interactions. Phosphorylated residues from Nup159 and interacting residues from Atg8 are highlighted in licorice. (D) Average number of heavy atom contacts (Av. num. of contacts; distance lower than 5 Å) per frame between potentially phosphorylated Nup159 residues and Atg8 residues. Data from triplicate (1 µs each) MD simulations for five different systems: unphosphorylated from the predicted WT structure (SER/THR), unphosphorylated from the predicted phosphomimetic structure (SER/THR-helix), with phosphomimetic mutations (GLU), and phosphorylated (SP1/TP1: -HPO_4_^*−*^ and SP2/TP2: -PO_4_^2*−*^, respectively).

Using the Atg8-Nup159 AIM1 system as an example, we analyzed the structural effect of the phosphomimetic mutations. We compared the predicted structures from all fragments that formed a canonical LC3-LIR interaction with Atg8 for the WT (Figure 6A) and the phosphomimetic (Figure 6B) Nup159 fragments, respectively. The core LIR residues and their C-terminal region behaved similarly. In the N-terminal region, the majority of the phosphomimetic fragments formed a short α-helix that was not observed for the wild type. This short helical stretch bound in a groove at the surface of Atg8. We hypothesized that this helix positions the phosphomimetic Nup159 residues in such a way that they can interact with Atg8. A shorter (one turn) helix N-terminal to the core LIR has been previously described in the structure of a fusion construct between GABARAP and TEX264’s (testis expressed 264, ER-phagy receptor) LIR_273–276_ with a phosphomimetic S272D mutation [57], which would correspond to the S1077E mutation in our predictions (Figure S6A). However, in a structure with p-S271 and p-S272 this helix does not form [57]. To probe the stability of the predicted structural elements, and the interactions formed by the potential phospho-sites we turned to molecular dynamics (MD) simulations. This also allowed us to introduce phosphorylated residues and compare their interactions and structural impacts to those of the phosphomimetic glutamates.

We investigated the interactions formed by the phosphorylatable residues in MD simulations of the Atg8-Nup159 system in different phosphorylation states. We extracted a representative structure of a 20-residue long Nup159 fragment centered on AIM1 (by truncation of a longer fragment) and bound to Atg8 for the WT and phosphomimetic system, respectively. For the phosphomimetic system, we also replaced the phosphomimetic residues with WT residues and with unprotonated (SP2 and TP2 with charge *−*2) or singly protonated (SP1 and TP1 with charge *−*1) phosphorylated residues. Hence, we ran simulations of five different systems: WT from WT structure (SER/THR), WT from ST structure (SER/THR-helix), ST from ST structure (GLU), SP1/TP1 from ST structure (SP1/TP1), and SP2/TP2 from ST structure (SP2/TP2). In all simulations, the core LIR stayed stably bound to HP1 and HP2 of Atg8, while the *N*- and C-terminal segments were more dynamic. This includes the short N-terminal helix, which partially unfolded in a few simulations with phosphomimetic or phosphorylated residues. Notably, while the helix was also mostly stable once WT residues were reintroduced, this system showed consistent unfolding at the very C terminus of the helix containing the phospho-sites S1075 and S1077 (Figure S6B).

We found that phosphomimetic and phosphorylated Nup159 residues formed similar contacts with Atg8, interacting mainly with a few positively charged residues. These residues were mainly Arg20, Arg24, Lys46, Arg47, and Lys48 for the three N-terminal phospho-residues p-S1073, p-S1075, and p-S1077. For the C-terminal p-T1086, the main interaction partner was Arg28, though we also observed some interactions with Arg65 and Lys66 (Figure 6C,D). The general pattern of interactions was similar for phosphomimetic and phosphorylated residues, with the strength of the contacts increasing as E *<* SP1/TP1 *<* SP2/TP2. By contrast, for the unphosphorylated residues, only S1077 (and to a limited degree S1075 in the WT simulation starting from the phosphomimetic structure) showed a similar pattern, while the other residues preferred different interaction partners when compared to their phosphorylated counterparts.

### Further LC3-LIR interactions from screen of LC3 proteins

We investigated the influence of the chosen LC3 protein on the predictions by AlphaFold2. Since the binding affinity between a LIR and an LC3 has been shown to vary by up to an order of magnitude for different LC3s [58], we reasoned that AlphaFold2’s predictions might reflect this. Hence, we applied our pipeline to three proteins containing LIRs with varying affinity for LC3B and GABARAP (Figure S7).

**Figure 7.**
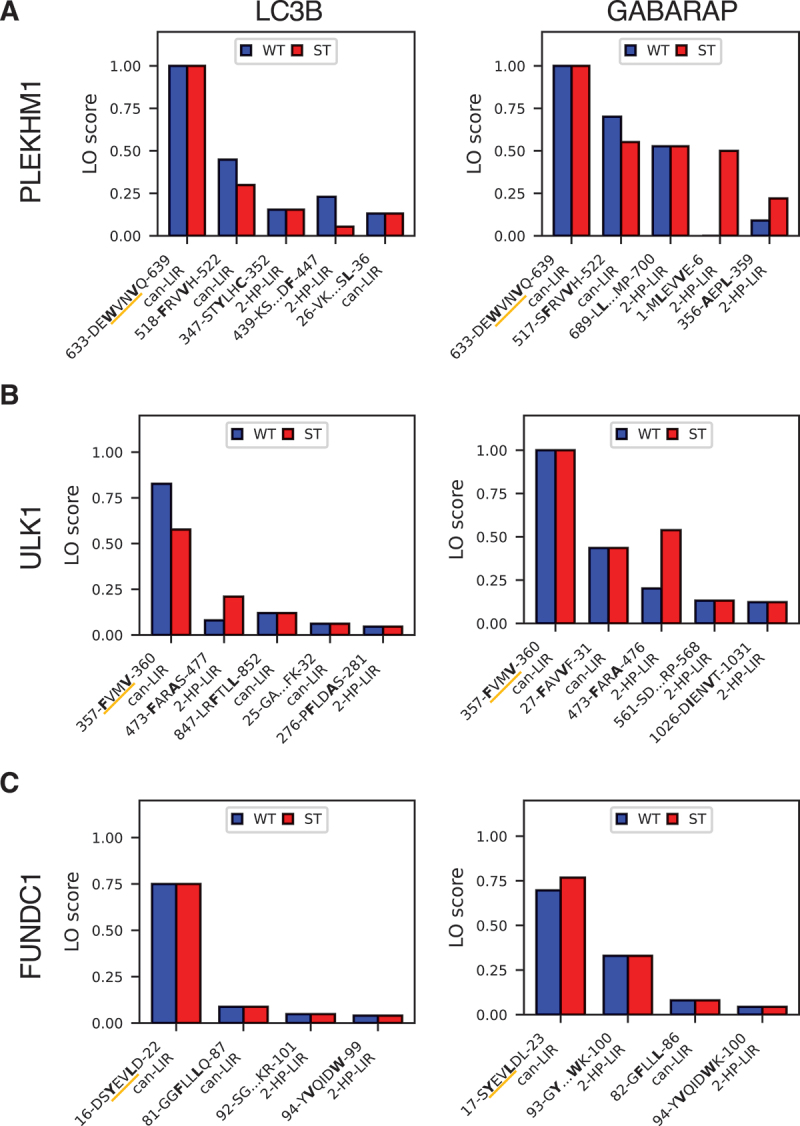
The prediction of LC3-LIR interactions depends on the LC3 protein. Results of fragment-prediction screens for (A) PLEKHM1, (B) ULK1, and (C) FUNDC1 combined with (i) LC3B and (ii) GABARAP. Shown are the top five (or all four for FUNDC1) predicted LIRs, sorted by LO score. Residues binding in hydrophobic pocket 0, 1, and 2 are highlighted in bold. The interaction type is indicated below each motif. The experimentally confirmed LIRs are underlined in orange.

For three examples (PLEKHM1 [pleckstrin homology and RUN domain containing M1], ULK1 [unc-51 like autophagy activating kinase 1], and FUNDC1 [FUN14 domain containing 1]), we found moderate differences between LIRs predicted in interactions with LC3B and GABARAP. For the experimentally confirmed LIRs in PLEKHM1 [59] and FUNDC1 [3,60], LIR_635*−*638_ and LIR_18*−*21_, respectively, we observed basically no difference (Figure 7A,C). By contrast, for ULK1’s LIR_357*−*360_ [27,53] we found a notable increase in fragments containing the interaction with GABARAP as compared to LC3B (Figure 7B). This is consistent with the experimental data showing that the difference in affinity for ULK1’s LIR_357*−*360_ is the largest for the three screened proteins [58]. Interestingly, for other predicted LIRs with overall weaker scores, the difference between GABARAP and LC3B was larger. Whereas GABARAP tended to give stronger signals overall, the LC3B signal was found to dominate for individual LIRs. One example is the candidate low-confidence LIR_440*−*442_ for PLEKHM1, which had scores of 0.67 (WT) and 0.47 (ST) for LC3B and scores of 0.57 (WT) and 0.04 (ST) for GABARAP. Overall, these results suggest that repeating the screen with different LC3 proteins may yield additional LIRs, and might indicate differences in affinity.

### Fragment-based AlphaFold2 scan finds canonical and non-canonical candidate LIRs in NUP214

We chose NUP214 (nucleoporin 214) as an application example. As the human analog to Nup159 [61], NUP214 may be targeted by LC3 proteins as well. However, its length of over 2000 residues makes it a difficult target for experimental and computational screens alike.

**Figure 8.**
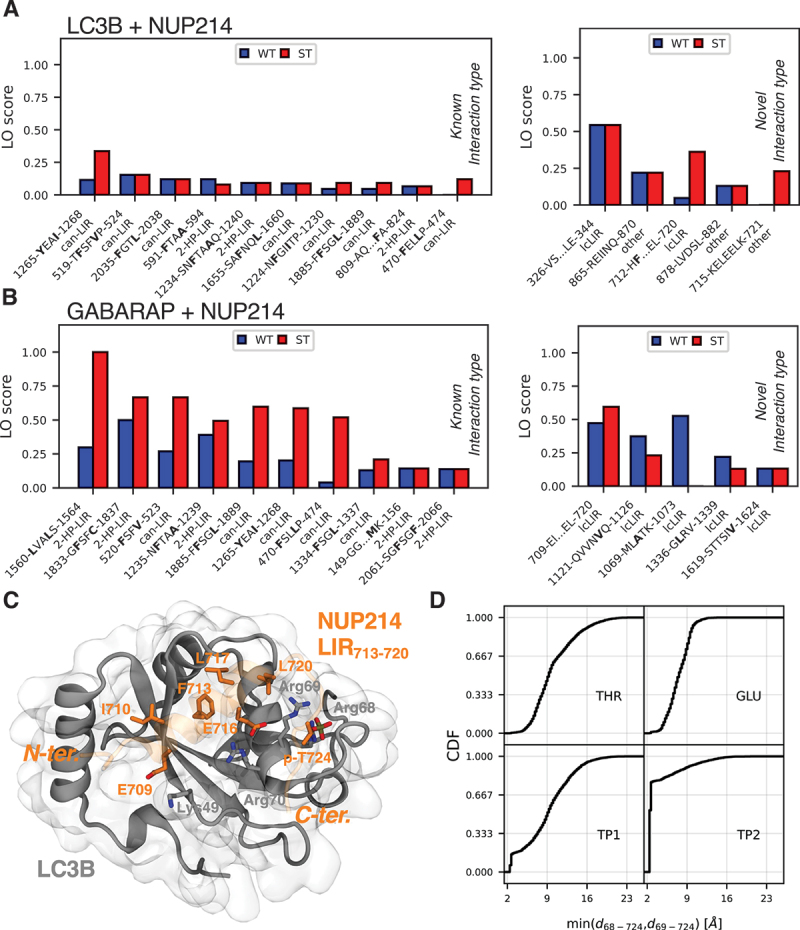
The fragment-based AlphaFold2 screen predicts multiple phosphorylation (in)dependent LIRs in NUP214. Results of fragment-prediction screens for NUP214 combined with (A) LC3B and (B) GABARAP. Shown are the top ten predicted LIRs with known interaction types and the top five predicted LIRs with novel interaction types, sorted by LO score. Predicted motifs with an average residue depth ≥ 0.3 nm are excluded. Residues binding in hydrophobic pocket 0, 1, and 2 are highlighted in bold, interaction type is indicated under the motif. (C) Snapshot structure of LC3B and a NUP214 fragment containing the low-confidence LIR_713*−*720_ with one phosphorylation after 1 µs of MD simulation. Interface forming residues from NUP214 and some selected residues from LC3B are highlighted in licorice. (D) Cumulative distribution functions (CDFs) for the minimum value of the distances between the potentially phosphorylated NUP214 residue T724 and Arg68 or Arg69 from LC3B, respectively. The two distances were computed for every timestep of the analysis and the lower one then added to the histogram. Steps below *≈*4 Å indicate the fraction of tightly coordinated structures in the MD simulations. Data from triplicate (1 µs each) MD simulations for four different phosphorylation states: unphosphorylated (THR), phosphomimetic (GLU) mutation, and phosphorylated (TP1: -HPO_4_^*−*^ and TP2: -PO_4_^2*−*^, respectively).

In our NUP214 fragment screen, we found more promising candidate LIRs with GABARAP (Figure 8B and S8A) than with LC3B (Figure 8A and S8A). Strong LIR candidates are found in a high fraction of the fragments and are not located in well-folded parts of the protein. Additionally, if they are similar to a previously described interaction this further increases confidence, but we also considered novel, low-confidence, interaction types. For the GABARAP screen, some of these strong candidates were sensitive to phosphomimetic mutations, including the ncLIR_1560*−*1564_ and the canonical LIR_520*−*523_, LIR_1885*−*1889_, and LIR_1265*−*1268_. This last LIR was also a relatively strong and phosphorylation-dependent candidate in the LC3B screen and is predicted also by iLIR [30]. Another strongly phosphorylation-dependent candidate in the LC3B screen was the low-confidence LIR_713*−*720_, which appeared as LIR_709*−*720_ also in the GABARAP screen.

To further probe these LIR candidates and the stability and interactions in the predicted structures, we used MD simulations without and with phosphorylation. Similar to the Atg8-Nup159 AIM1 system, we found that phosphomimetic and phosphorylated NUP214 residues in LIR_1265*−*1268_ interacted mainly with a few positively charged residues of LC3B and GABARAP, respectively. For the interaction with LC3B, we observed identical trends to the Nup159 system. In particular, the general pattern of interactions was similar for phosphomimetic and phosphorylated residues, with the strength of the contacts increasing from E *<* SP1 *<* SP2 (Figure S8B). However, this trend was less clear for GABARAP, with notable differences in the interactions of SP1 and SP2 (Figure S8C). Also, the phospho-residues interacted with different residues in LC3B and GABARAP. For example, p-S1257 interacted with Arg10 in LC3B, but in GABARAP Glu8 is located at this position and did not interact with p-S1257. This suggests that while the general nature of these interactions is similar, they depend on the LC3 protein. The role of residues that are “charge-swapped” between LC3B and GABARAP has been discussed previously [62] and phosphorylation might add an additional layer contributing to LC3 specificity.

LIR_713*−*720_ formed an α-helix that dynamically interacted with LC3B via a hydrophobic interface and p-T724. In the MD simulations, the predicted helical LIR segment stayed bound to LC3B for 1 µs independent of T724’s phosphorylation state (Figure S8D). It formed a hydrophobic interface with LC3B with the residues I710, F713 (bound HP2), L717, and L720. E709 and E716 further stabilized this interface by interacting with Lys49 and Arg70 (Figure 8C). However, the hydrophobic interface was quite dynamic, with the helix displaying varying conformations and partial unbinding (Figure S8D). Replacing T724 by a phosphomimetic or phosphorylated residue strengthened the interaction with Arg68 and Arg69. In the fully deprotonated state (TP2 with charge *−*2e), p-T724 was firmly anchored to these residues (Figure 8D). The dynamic and not completely stable binding of the helix, and the firm anchoring of p-T724 suggest a strong phosphorylation dependence of this interaction, consistent with the strong dependence of the AlphaFold2 prediction on phosphomimetic mutations for this candidate LIR.

### Fragment-based AlphaFold2 screen of SLiM-mediated interactions

**Figure 9.**
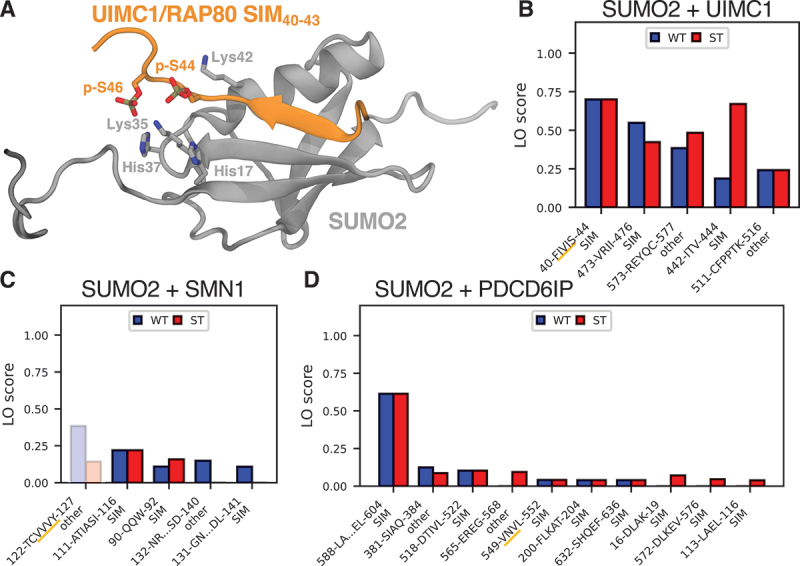
Fragment-based predictions may be suitable for other protein-protein interactions involving short linear motifs, including SUMO-SIM interactions. (A) Previously determined NMR structure of the UIMC1/RAP80-SIM bound to SUMO2 (PDB ID: 2N9E) [65]. Phosphorylated residues from UIMC1 and interacting residues from SUMO2 are highlighted as licorice. Results of fragment-prediction screens for (B) UIMC1, (C) SMN1, and (D) PDCD6IP combined with SUMO2. Shown are the top five (ten for PDCD6IP) predicted interacting motifs (both SIMs and others), sorted by LO score. The interaction type is indicated below each motif. The experimentally identified SIMs are underlined in orange. Predicted motifs with an average residue depth ≥0.3 nm are faded out.

We tested the transferability of our method to other interactions mediated by SLiMs, focusing on SUMO-SIM interactions as an example. Similar to canonical LIRs, SIMs are SLiMs and their binding to SUMO proteins can be modulated by phosphorylation [38,63]. Hence, we reasoned that a fragment-based screen should be useful to identify potential SIMs. As poof of principle, we studied the complex between SUMO2 and the SIM_40*−*43_ of UIMC1/RAP80 (ubiquitin interaction motif containing 1) [64] (Figure 9A).

Screening UIMC1 fragments with and without phosphomimetic mutations returned the experimentally confirmed SIM with high consistency (Figure 9B). Additionally, there are two more SIMs predicted with high consistency: SIM_442*−*444_ and SIM_473*−*476_. Interestingly, the experimentally confirmed SIM_40*−*43_ and SIM_473*−*476_ both follow the canonical Γ-Γ-X-Γ/Γ-X-Γ-Γ SIM motif, where Γ is I, V or L, and X any amino acid [37], and their prediction was not – or only very weakly – influenced by phosphomimetic mutations. By contrast, the prediction of the candidate non-canonical SIM_473*−*476_ strongly depended on these mutations. The experimentally confirmed SIM and the two strong candidates are all located in relatively unstructured regions of the protein that are accessible to interactions (Figure S9A).

For two other proteins, finding the experimentally identified SIMs proved more challenging. For SMN1/SMN (survival of motor neuron 1, telomeric) and PDCD6IP/ALIX (programmed cell death 6 interacting protein), SIMs have been identified via mutation studies [66,67]. However, no complex structure between the respective SIM and SUMO2 was determined, making them interesting targets for our computational screen. For both systems, the fraction of fragments containing predicted SIMs was noticeably lower than for UIMC1. While the experimentally identified SIM_124*−*127_ was the strongest signal we found for SMN1 in an unfiltered screen, it was not detected when filtering for a maximum average r.d. of 0.3 nm (Figure 9C). For PDCD6IP, the experimentally identified SIM_548*−*552_ was only at the 5th position (Figure 9D). Strikingly though, for SMN1 and PDCD6IP the (additional) top scoring SIM candidates were all in close proximity to the ones experimentally confirmed by mutations. It is also notable that for both SMN1 and PDCD6IP the experimentally identified and the strongest predicted SIM candidates lie within or at the edge of folded domains. Still, the low residue depth for these motifs (with the exception of SIM_124–127_ in SMN1) suggested some degree of accessibility (Figure S9B and C). Looking at the predicted structure of an SMN1 fragment containing SIM_124–127_ in complex with SUMO2 revealed some interesting nuance: AlphaFold2.3 predicted the motif to be the central β-sheet in a small, folded domain that bound SUMO2 at a different site than the predicted SIM-binding site (Figure S9E). The motif is also found to be interacting more prominently in longer fragments (Figure S9D).

## Discussion

The identification of functional – canonical and non-canonical – LIRs is a critical step in the identification of cargo and cargo receptor proteins in selective autophagy. Computational methods are powerful tools to design and guide experimental studies aimed at identifying new LIRs. Here, we investigated the feasibility of a fragment-based approach that makes use of the predictive power of AlphaFold2 to find novel LIR candidates via the prediction of LC3-LIR complexes (results summarized in Table S1).

We reasoned that a fragment-based screen could be an alternative or complimentary approach to a full-length prediction. Obvious advantages of such an approach are its ability to find multiple LIRs for a sequence (e.g., for Nup159, Figure 3F), and hidden LIRs, which would be (partially) buried in the complete structure and become accessible only upon (local) unfolding (e.g., for CALR, Figure 1C,D). Possible disadvantages are a likely higher number of false positives, in particular of buried LIRs that are unavailable for interaction in the protein in functional conformations. This problem can be addressed by including structural context like residue depth and secondary structure, since reasonable models of the full-length structures (of only the protein, not the complex) are readily available thanks to AlphaFold2 and 3. Still, fragmentation decreases the chance of finding discontinuous LIRs, and due to the multiple predictions needed for a single protein, a fragment-based screen is also computationally more expensive. Despite these drawbacks, the example of CALR highlights quite nicely that the additional sensitivity of this approach is essential in some cases. Also, once very long proteins are involved, full-length complex structure predictions become difficult or even unfeasible. Combined, this results in a range of cases in which a fragment-based approach is preferable to a full-length prediction.

The length of the fragments plays an important role in the search for LIRs and other SLiMs. Altering the length of fragments may be inconsequential in some cases, e.g., for the LC3B-OPTN system. In other cases, e.g., the Atg8-Nup159 system, fragment length has a strong effect on the quality of the prediction. Furthermore, in our sequence scans we found that shorter fragments tend to produce notably more protein complex structures than longer ones. This indicates a possible trade-off between false positives and false negatives for short and long fragments, respectively. However, this relation does not hold strictly true in all cases, and some interactions are only picked up in longer fragments, most notably for the SUMO2-SMN1 system. Strikingly, in this example the predicted structures suggest that the interacting motif may not actually be a SLiM but rather part of a small, folded domain that interacts with SUMO2. Repeating the screen with various fragment lengths offers a good compromise between sensitivity and specificity, albeit at increased computational cost.

The phosphomimetic mutations we introduced seem to have a mixed effect on the sensitivity in the prediction of LC3-LIR interfaces. Phosphomimetic mutations did not meaningfully influence the prediction of experimentally confirmed LIRs in many of the tested cases, e.g., for OPTN, CALR, PLEKHM1, ULK1, and FUNDC1, and in some cases even decreased sensitivity, e.g., for the sAIMs in C53 (*A.t*.). However, they played a critical role in finding Nup159’s AIM1 (Figure 3F), and many of the novel LIR or SIM candidates identified in, e.g., ULK1, NUP214, and UIMC1. Interestingly, binding of OPTN’s LIR to LC3B has been shown to be strongly phosphorylation dependent in experiments [25]. This indicates that while the effect of phosphomimetic mutations on the predicted interaction might serve as an indication for true phosphorylation dependence, it may not show up in the screen because the prediction is already at a very high confidence for the wild type. Since we also observed an increase in scores for confirmed nonfunctional LIRs (Figure 4), it should be noted that the increased sensitivity seems to come at the cost of decreased specificity.

It should be noted that structures are available for some well-performing cases, which may appear in AlphaFold2’s training set. While the screen does perform extremely well for these cases, it does also yield good results for LIRs for which the structure is not yet solved, e.g., for ULK2, STBD1, and AIM1_1078* − *1081_ in Nup159. Furthermore, the confidence of the prediction in some of the novel candidates rivals the one for the structurally confirmed LIRs. The case of CALR highlights that even with a structure for the interaction being available, it is not guaranteed that AlphaFold2 will predict it given the full-length protein sequences. Altogether, we argue that the screen is capable of not only reproducing existing structures, but also of generating high-confidence predictions of novel LIR candidates.

Notably, in many cases where the screen produced only weak results for experimentally confirmed interactions, it found stronger signals close by, e.g., for GABARAP-CDK5RAP3 (*H.s*.) and SUMO2-PDCD6IP. On the one hand, these signals may just be false positives, and indicate the limitations of this method. On the other hand, there are two additional interpretations: First, the experimental results for these systems might be incomplete, as they are based on mutation studies without structural evidence. Hence, it is possible that these mutations do not lie in the actual SLiM but indirectly affect the interaction with the proximal true interface. Second, the identified motifs may offer interactions in addition to the experimentally identified ones that lead to an increase in avidity and may also increase specificity [68]. This is especially intriguing for proteins binding SUMO2, since it forms poly-SUMO2 chains in multiple cellular contexts. These SUMO2 chains provide the complementary multivalent binding interfaces for proteins with multiple SIMs [69]. Also, it has been recently proposed that in the context of poly-SUMO multivalency, auxiliary interactions not involving SIMs indeed play a role [70]. Similar to SUMO chains, one can think about multiple LC3s attached to a phagophore as multivalent binding spots for proteins with multiple LIRs, as has been discussed previously [71].

As a curiosity, we found that some SLiMs, which had been previously confirmed as functional by mutating them in experimental studies [54,66], are predicted by AlphaFold2 to form part of the core of folded domains. Most notably, this applies to DSK2A’s LIR_43–46_ and SMN1’s SIM_124–127_ (Figure S5B and S9E). However, this may be explained by various factors, such as the limitations of AlphaFold2 or these being barely stable domains that (partially) unfold under physiological conditions. On a more speculative note, assuming a correct prediction by AlphaFold2 and stably folded domains, these domains may interact with LC3s and SUMOs, respectively, not as SLiMs but via a different interaction mode, e.g., as shown for a SUMO2-SMN1 prediction in Figure S9E. The mutations meant to disrupt the respective SLiMs could then instead lead to an un- or misfolding of these domains, impairing the interaction with LC3/SUMO that way.

The screen is also capable of picking up differences in the interaction between a LIR and different LC3s if they are large enough. PLEKHM1’s and ULK1’s experimentally identified LIRs have been found to interact more strongly with GABARAP [58]. Our screen picked this up for ULK1, where the difference in binding affinity is the largest. For FUNDC1’s LIR_18*−*21_, a slight preference for LC3B is expected, but not picked up by our screen. Similarly, the slight preference of PLEKHM1’s LIR_635*−*638_ for GABARAP is not picked up. For NUP214, we found more strong signals when screening against GABARAP than when screening against LC3B, though individual signals, e.g., for the low-confidence LIR_326–344_ can be stronger for LC3B. Overall, screening against GABARAP tends to generate more strong-signal candidate LIRs.

We used MD simulations to investigate the interactions of phosphorylations in LIR binding. For the two canonical LIR motifs we simulated, AIM1_1078*−*1081_ in Nup159 and candidate LIR_1265*−*1268_ in NUP214, basic residues interact with the phospho-sites. Interestingly, the interaction pattern in the simulations of GABARAP with NUP214’s LIR_1265*−*1268_ deviates slightly for the singly protonated SP1 system, highlighting some strong interactions with Leu55, Thr56, and Gln59. Visual inspection revealed that these are formed by H-bonds between the phosphate group of residue p-S1271 and the backbone and sidechains of Thr56 and Gln59. Notably, phospho-residue interactions with most of the mentioned positively charged residues (or their analogs in other LC3 structures) have been described previously for other LC3-LIR interactions [25,26,72]. The one lcLIR we simulated, candidate LIR_713*−*720_ of NUP214, contains only one phosphorylation, which interacted strongly with Arg68 and Arg69 of LC3B. Additionally, residues Lys49 and Arg70 formed interactions with acidic residues flanking the hydrophobic LC3-LIR interface. For Nup159 AIM1, AlphaFold2.3 predicted the formation of a short N-terminal helix in the phosphomimetic but not the WT system. In our simulations, this helix was similarly metastable in phosphomimetic and phosphorylated systems. By contrast, previous findings suggest that the structure of some bound LIRs differs between phosphomimetic and phosphorylated constructs [57]. While this may be explained by the different LC3-LIR systems or shorter LIR fragments in the experiments (4 or 2 residues N-terminal of Θ, compared to 8 in our simulations), the limitations (especially timescales) of MD simulations may be another explanation. For all LC3-LIR interactions we simulated, the additionally formed contacts of phosphomimetic and phosphorylated residues are consistent with the increased fraction of fragments that contain the predicted LIR, once phosphomimetic mutations for selected serine and threonine are introduced in the AlphaFold2 screen.

We did not include phosphomimetic mutations of tyrosines for two reasons. Like serine and threonine, tyrosines are also commonly phosphorylated, and have also been shown to modulate LC3-LIR interactions [3]. However, since tyrosines can serve as the Θ residue for LIRs, phosphorylated tyrosines are more likely to have a negative effect on LC3-LIR interactions, making them less useful in the identification novel LIRs. Additionally, while phosphomimetic mutations of serine and threonine to glutamate are structurally similar, for tyrosines the changes would be quite substantial. Still, if one’s priority is to assess the effect of phosphorylation on the interaction, tyrosine phosphorylation should be considered, e.g., by including only modifications for residues targeted by one specific kinase. Here, very recent developments allowing the explicit and direct consideration of PTMs in protein structure predictions, as in the recently published AlphaFold3 [43], should prove valuable.

### Recommendations for practitioners

In LIR-screens with limited compute budget, we recommend to extend the screen to the full-length target protein, to 36mer fragments of the relevant regions of the target protein, and then to repeat the process with phosphosites (S, T) changed to phosphomimetic E. As outlined in Figure S2C and supported by the results in Figure 3A–F and S3A-C, this fragment length offers a good compromise between specificity and sensitivity at limited computational cost. By testing their accessibility in experimental or AlphaFold structures of the intact target protein, the list of candidate LIRs can be further trimmed. It is also possible to assess the full structure in advance and exclude well-folded segments from the screen to reduce computational cost. However, this may leave partially buried LIRs, as in CALR, undiscovered. A detailed examination of the predicted structures for strong candidates and of the AlphaFold indicators of confidence in the predicted interactions then gives further insights into the expected strength of the LIR predictions obtained from the computational screen and a basis to select peptides and mutation sites for experimental validation.

## Concluding remarks

With AlphaFold3 now being available both as an online server and as source code including model weights [43], we expect further future improvement of our pipeline. Among its novel features, the possibility to include real PTMs in the structural predictions makes not only phosphotyrosines more accessible, but also allows the inclusion of, e.g., acetylation. Thus, one can include a greater variety of PTMs that have been shown to modulate LC3-LIR [71] and SUMO-SIM [73] interactions. Also, we observed a general improvement of AlphaFold2’s predictions between versions 2.2 and 2.3, and also – though still anecdotal – between versions 2.3 and 3. A continuation of this trend would be welcome both for fragment-based but also for full-length-based LIR predictions. With the source code now available, AlphaFold3 can be used for high-throughput peptide screens. Besides AlphaFold3, there are also other approaches on the way to better account for phosphorylation in AlphaFold2-based predictions [74]. Additionally, our approach can easily be adapted for other structure prediction methods, such as OpenFold [75] and RoseTTAFold2 [76], though we did not test how this will affect results. Lastly, as an increased number of non-canonical LC3-LIR structures will become available in the future, one can expect the quality of predictions of these complex structures to improve further.

## Methods

### Fragment sampling pipeline

#### Generation of fragment sequences

We employed two different schemes for the generation of fragment sequences, one to investigate the effect of fragment length and another to screen over an entire protein sequence. For the effect of fragment length, we chose a 4-residue core LIR as the minimum sequence. We increased the length in 2-residue steps (+1 at each terminus) up to a final length of 68 residues (32 N-terminal residues, 4 core LIR residues, 32 C-terminal residues). For the screening over protein sequences, we generated fragments of a defined length (52mers, 36mers, and 16mers) that jointly cover the entire protein sequence with 75% overlap. E.g., for 16mers, the first fragment contained residues 1–16, the second 5–20, the third 9–24, etc. For the last fragment, the starting residue was shifted, if necessary, to achieve the required length. We took sequences for all proteins from UniProt [77] (Table 1), and experimental phosphorylation sites from dbPTM [47] (with the exception of MEFV and TBC1D2, for which we used the PTM data from UniProt). For sequences, for which no large-scale data on experimental phosphosites were available, we assumed all S and T residues to be potential phosphosites. If a fragment did not contain any phosphosites, we used the results from the WT prediction also for the phosphomimetic sequence.

**Table 1.** Protein sequences used in this study.

Protein name	UniProt ID
Atg8	P38182
ATG8CL	M1C146
ATG8E	Q8S926
MAP1LC3B/LC3B	Q9GZQ8
GABARAP	O95166
OPTN	Q96CV9
CALR	P27797
Nup159	P40477
Joka2	M1BJF6
BNIP3	Q12983
STBD1	O95210
ULK2	Q8IYT8
MEFV/pyrin	O15553
TBC1D2/TBC1D2A	Q9BYX2
DSK2A	Q9SII9
CDK5RAP3/C53 (*Homo sapiens*)	Q96JB5
AT5G06830/C53 (*Arabidopsis thaliana*)	Q9FG23
PLEKHM1	Q9Y4G2
ULK1	O75385
FUNDC1	Q8IVP5
NUP214	P35658
SUMO2	P61956
UIMC1/RAP80	Q96RL1
SMN1/SMN	Q16637
PDCD6IP/ALIX	Q8WUM4

## Fragment and LC3 complex structure prediction

We used AlphaPulldown (versions 0.22.3 and 0.30.7 with AlphaFold2.2 and 2.3, respectively) [78] for the high-throughput AlphaFold2 Multimer [32,79] predictions. The only default setting we changed was the number of cycles (ncycles), which we increased to ten. We set the “max_template_date” parameter to “2050-01-01”. We treated every fragment as an individual protein, meaning we calculated a multiple sequence alignment (MSA) for every single one. If not stated otherwise, AlphaFold2 refers to version 2.3.

## Evaluation of generated structures

We evaluated the generated structures in two steps. The first step differed between the scans of fragment length and over entire sequences. For the length scans, we calculated the AlphaFold2 confidence scores for the four core LIR residues the respective scan was centered on. When scanning over the entire protein sequence, we looked for any interface that AlphaFold2 predicted with confidence. In the second step, we classified the confidently predicted structures based on the type of interaction between LC3 (or SUMO) and the respective fragment.

We used AlphaFold2’s “predicted local-distance difference test” (pLDDT) score and predicted aligned error (PAE) to quantify AlphaFold2’s confidence in its prediction. For each individual residue, we used the pLDDT score as given by AlphaFold2 and calculated a minimum PAE (minPAE) as the average of the three lowest PAE values when the respective residue was used for scoring (but not aligning). We looked for stretches in fragments, which fulfilled three criteria: (i) a minimum length of 4 residues, (ii) a minimum average pLDDT of 75 over all stretch residues, and (iii) a maximum average minPAE of 8.0 Å.

We classified LC3-stretch interactions based on their structure either as “canonical LIR” (can. LIR), “non-canonical LIR” (ncLIR, with multiple subclasses), “low-confidence LIR” (lcLIR), “other interaction proximal to the LIR-docking site” (other@LDS) interacting via an “ubiquitin-interacting motif-like” (UIM-like) sequence, or “other”. For the classification of canonical LIRs, we used four criteria: (i) Binding of a residue to HP1, (ii) binding of a residue to HP2, (iii) two or more backbone H-bonds with the LC3 β2-strand between HP1 and HP2, and (iv) the stretch having the canonical LIR sequence Θ-X-X-Γ—where Θ is W, F or Y, and Γ is L, I or V – with Θ having to be the residue bound in HP1 and Γ the one bound in HP2. Only if all four of these criteria were fulfilled, we classified a stretch as canonical LIR. If the stretch satisfied one to three (but not all four) of these criteria, we classified it either as ncLIR or lcLIR. For the classification as ncLIR, we compared the associated predicted structure to ncLIRs with at least some structural characterization. We checked for antiparallel LIRs (ap. LIR) [23], LIRs engaging HP0 (HP0-LIR) [19], LIRs with non-canonical residues engaging HP1 and/or HP2 (2-HP-LIR) [15], CLIRs [17,18], shuffled LIR/AIMs (sAIM) [16,80], and TRIM5-like LIRs ([DE]W[DE]-LIR]) [20], in that order (so a ncLIR matching multiple of these criteria will be classified as the first one it matches). We applied criteria that ensured similarity with the previously described motifs but did allow for some deviation. Antiparallel LIRs had to fulfill criteria (i) to (iii) and match a reversed canonical sequence of Γ-X-X-Θ. HP0-LIRs had to engage HP0 with Θ and HP2 with Γ and follow a Θ(HP0)-X-Γ-X-X-Γ(HP2) motif. We did not require HP1 to be engaged. A 2-HP-LIR had to fulfill criteria (i) and (ii) but could use any residues to engage HP1 and HP2. CLIRs had to fulfill criteria (ii) and (iii) and follow a Γ/M-Γ/M-Γ(HP2) motif. sAIMs had to follow a Γ-X-Θ-X motif and engage either HP0 or HP1 with Θ, since a recent study using NMR spectroscopy suggests involvement of HP0 in addition to HP1 and HP2 in sAIM binding [80]. A [DE]W[DE]-LIR had to follow a D/E-W-D/E motif and engage HP1 with W. Any motif fulfilling at least one of the four criteria for a canonical LIR but not matching the classification for canonical or ncLIR was classified as a speculative/novel lcLIR.

We determined binding to HP0, HP1 and HP2 by evaluating all residues from N to C terminus of the stretch until we found one that fulfilled certain distance constraints. We considered a residue bound to HP0 if the distance between any of its heavy atoms and two defined LC3 C_α_ atoms was below a given threshold, and the HP0 pocket was open in the structure (as indicated by the position of a specific residue N-terminally of the β2-strand [19]). The first distance to the C_α_ atom of residue 7 (LC3B and ATG8E)/6 (ATG8CL)/5 (GABARAP and Atg8) had a cutoff of 4.50 Å, the second distance to the C_α_ atom of residue 34 (LC3B and ATG8E)/33 (ATG8CL)/32 (GABARAP and Atg8) had a cutoff of 6.25 Å. For the opening of HP0, we looked at the minimum distance between the C_α_ atom of residue 7 (LC3B and ATG8E)/6 (ATG8CL)/5 (GABARAP and Atg8) and any non-hydrogen sidechain atom of residue 50 (LC3B)/49 (ATG8E)/48 (ATG8CL)/47 (GABARAP and Atg8). If this distance was larger than 9.0 Å we considered HP0 to be open. We considered a residue bound to HP1 if the distance between any of its heavy atoms and two defined LC3 C_α_ atoms was below a given threshold. The first distance to the C_α_ atom of residue 52 (LC3B)/51 (ATG8E)/50 (ATG8CL)/49 (GABARAP and Atg8) had a cutoff of 5.25 Å, the second distance to the C_α_ atom of residue 108 (LC3B)/106 (ATG8E)/105 (ATG8CL)/104 (GABARAP and Atg8) had a cutoff of 9.75 Å. For HP2, we used a similar approach with two different respective distances. The first distance to the C_α_ atom of residue 54 (LC3B)/53 (ATG8E)/52 (ATG8CL)/51 (GABARAP and Atg8) had a cutoff of 4.75 Å, the second distance to the C_α_ atom of residue 67 (LC3B)/66 (ATG8E)/65 (ATG8CL)/64 (GABARAP and Atg8) had a cutoff of 9.50 Å.

We used the dssp [81] definition with the default cutoff of *−*0.5 kcal/mol to determine H-bonds. Since the structures generated with AlphaPulldown lacked hydrogen atoms, we added backbone hydrogens in post-processing via PyMol’s h_add [82] method. We calculated the number of H-bonds between all residues of the confidently predicted stretch and the LC3 β2-strand between HP1 and HP2: residues 50–55 for LC3B, residues 49–54 for ATG8E, residues 48–53 for ATG8CL, and residues 47–52 for Atg8 and GABARAP.

All stretches without a LIR were classified as “other interaction proximal to the LIR docking site” if they formed at least five C_α_ atom contacts (distance below 5 Å) with the LC3 β2-strand between HP1 and HP2. Stretches with at least five C_α_ atom contacts with the LC3 UIM-like docking site (UDS) (residues 79–82 for LC3B, residues 78–81 for ATG8E, residues 77–80 for ATG8CL, and residues 76–79 for Atg8 and GABARAP) received a “UIM-like” classification. All remaining stretches were classified as “other”.

For SUMO2-SIM interactions, we made no distinction between canonical and non-canonical SIMs. We counted backbone H-bonds between the β-sheet of SUMO2’s SIM binding groove (residues 29–34) and the interacting confidently predicted stretch. For two or more H-bonds, we classified the interaction as a SUMO-SIM interaction. All other interactions we summarized as “other”.

## Estimation of residue occlusion

We used residue depth with respect to the protein surface and local secondary structure as quantifiers of residue occlusion in structures of the full-length target sequence. To get full-length structures we used AlphaFold2 (see section “AlphaFold2 predictions of full-length sequences”). We calculated residue depth via msms and BioPython’s ResidueDepth module [48,49], and secondary structure via dssp [81]. We only included parts of the structure in the residue depth (r.d.) calculation that had a pLDDT score of at least 70. We handled the resulting fragmentation by spawning additional probes at the N and C terminus of each resulting fragment. In two cases, for MEFV and TBC1D2, we only used the part of the structure with a pLDDT of 90 or higher, since a cutoff of 70 resulted in extensive fragmentation leading to unreliable results. When calculating the average residue depth of a motif, we used a value of 0.142 nm for residues with unassigned residue depth. We obtained this value from calculating the residue depth of a single glycine residue after 500 steps of energy minimization in vacuum using Gromacs (version 2022.4) [83] and the charmm36m force field (July 2021 version) [84]. Since we used the average residue depth as an upper cutoff, we assumed this lowest possible value for residues with no confidently predicted structure. Additionally, as has been discussed previously, low pLDDT scores in AlphaFold2 strongly indicate structural disorder [85] further justifying the use of a minimum residue depth value for these residues. We used the simplified definition for structural elements and required a residue pLDDT of at least 70 to assign secondary structure.

## Summary of predicted interaction motifs

We grouped stretches predicted in different fragments of varying length with and without phosphomimetic mutations together, if they predicted the same interaction type (e.g., canonical LIR or antiparallel LIR), engaged the same HPs with the same residues, and had an overlap of at least 3 residues. We ordered the summarized predictions by the relative occurrence of the interaction, meaning the number of fragments that contain the sequence stretch and form the interaction divided by the number of all fragments containing it. We calculated this property for each fragment length and mutation state individually. We ordered the predicted interacting stretches by the sum of this relative occurrence score for a given fragment length, giving longer fragments priority over shorter ones (52mers *>* 36mers *>* 16mers).

## LIR scores

We tested three metrics to summarize the predictions across different fragment lengths: best core-motif pLDDT, best core-motif minPAE, and length-weighted fraction of occurrence (LO) score. To obtain the best core-motif pLDDT we averaged the pLDDT scores of the 4 highest scoring consecutive residues within the motif for every fragment containing it in the interaction mode of the peak. We used the highest average value of all fragments as best core-motif pLDDT. We used a similar approach to obtain the best-core motif minPAE, this time averaging over minPAEs and taking the lowest one. The LO score was calculated as the sum over the fragment length of all fragments containing the peak motif and forming the respective interaction divided by the sum of all fragments containing the motif.

To calculate the distance of impactful phosphomimetic mutations from the motif it affected, we compared WT and ST fragments pairwise for every peak. For every peak in a fragment of one modification state (WT or ST), we tested if there was a corresponding peak in the fragment covering the same part of the sequence in the respective alternative modification state. For instance, if a peak occurred in a WT fragment covering residues 5–20, we would check if a similar peak (same similarity definition as used for grouping) existed in the ST fragment covering residues 5–20. If this was not the case, we considered the mutation to be impactful and calculated its distance to the motif of the peak. Hence, a phosphomimetic mutation was deemed impactful, if it either changed the interaction type (e.g., from canonical LIR to lcLIR) or moved the peak below/over the detection cutoff (from over/below in the WT sequence, respectively). If no fragment of alternative modification state existed for a peak, indicating that it did not contain mutation sites, it was discarded.

### AlphaFold2 predictions of full-length sequences

We used local installations of AlphaFold2.2 and AlphaFold2.3 Multimer, as well as the AlphaFold3 online server [43], to predict complex structures of full-length proteins from their amino acid sequence. For single proteins we used already predicted structures provided by the AlphaFold Protein Structure Database [86] if available, and AlphaFold2.3 otherwise. If not stated otherwise, AlphaFold2 refers to version 2.3.

### Molecular dynamics simulations

We used Gromacs (version 2023.2 for system preparation and equilibration, version 2023.3 for production runs) [83] and the charmm36m (July 2021 version) force field with an increased Lennard-Jones ϵ parameter for the water hydrogens for all molecular dynamics (MD) simulations [84]. We ran simulations of Atg8 in complex with Nup159 residues 1070–1089 (AIM1_1078*−*1081_), LC3B in complex with NUP214 residues 1257–1272 (LIR_1265*−*1268_), LC3B in complex with NUP214 LIR_1265*−*1268_, and GABARAP in complex with residues 703–728 (LIR_713*−*720_). All initial complex structures came from AlphaFold2 predictions in the context of the screen, with some being truncated. For AIM1_1078*−*1081_ and LIR_1265*−*1268_ systems we used predictions with WT sequences for WT simulations and predictions with phosphomimetic sequences for simulations of phosphomimetic/phosphorylated structures. For AIM1_1078*−*1081_ we also performed simulations of a WT system derived from the phosphomimetic structure by reversing the phosphomimetic mutations via a custom Python script. For LIR_713*−*720_ systems we used a prediction based on a phosphomimetic sequence also for WT simulations. We used charged termini for LC3 proteins and N-terminal acetylation and C-terminal aminomethylation capping groups for LIR-containing fragments. Phosphorylations were introduced into the structures with phosphomimetic mutations (replacing them) via a custom Python script. We solvated the systems in TIP3P water and added 150 mm NaCl plus neutralizing ions.

We minimized all systems using the steepest descent algorithm with maximum force for convergence of 1000 kJ mol^*−*1^ nm^*−*1^. Equilibration was done with one NVT and two NPT runs, all using a timestep of 1 fs, running for 1 ns, 1 ns, and 5 ns respectively. The NVT runs used a Berendsen thermostat [87] to maintain a temperature of 300 K with a characteristic time τ_T_ of 0.1 ps. The first NPT run used a Berendsen thermostat to maintain a temperature of 300 K with τ_T_ = 0.1 ps, and an isotropic Berendsen barostat [87] with a target pressure of 1 bar, a characteristic time τ_p_ of 5.0 ps, and a compressibility of 4.5*·*10^−5^ bar^−1^. The second NPT run used a v-rescale thermostat [88] to maintain a temperature of 300 K with τ_T_ = 1.0 ps, and an isotropic Parrinello-Rahman barostat [89] with a target pressure of 1 bar, a τ_*p*_ of 5.0 ps, and a compressibility of 4.5*·*10^*−*5^ bar^*−*1^. The minimization and the first two equilibration steps used position restraints on all protein heavy atoms.

Production runs were performed for 1 µs with a timestep of 2 fs. For temperature coupling we used a v-rescale thermostat with a target temperature of 300 K, and τ_T_ = 1 ps. For pressure coupling we used an isotropic Parrinello-Rahman barostat with a target pressure of 1 bar, τ_*p*_ = 5.0 ps, and a compressibility of 4.5*·*10^*−*5^ bar^*−*1^.

All simulations used a leap-frog integrator, a Verlet cutoff-scheme [90], a pair-distance cutoff (1.2 nm) with a force-switch modifier (1.0 nm) for Van-der-Waals interactions, a cutoff of 1.2 nm for Coulomb interactions, and Particle Mesh Ewald for long-range electrostatics [91]. Bonds for hydrogens were turned into constraints and treated with the LINCS algorithm [92].

We determined the average number of contacts for every residue pair by calculating the distances between their heavy atoms. If a pair distance was below 5 Å, the two atoms were counted as being in contact. We averaged over all frames (in 1 ns timesteps) of triplicate simulations. Plots show the 15 LC3 residues with the highest average number of contacts summed over the shown target residues. We calculated the secondary structure in simulation trajectories via dssp [81] in timesteps of 1 ns.

### Implementation and general analysis

We visualized all structures with VMD [93], which was also used for structural alignment and general analysis. Furthermore, we employed Python3 [94] with Anaconda3 [95], iPython [96], Numpy [97], Matplotlib [98], Seaborn [99], MDAnalysis [100], MDTraj [101], BioPython [49], and PyMOL [82] to implement the analysis described in the previous sections.

## Supplementary Material

SI_Stuke_R3.docx

## Data Availability

All data of this study (lists of all predicted LIRs, AlphaFold structures, MD simulation trajectories) are available from the authors upon reasonable request. The code used to setup the AlphaPulldown runs and analyze the resulting AlphaFold predictions is publicly available under https://github.com/bio-phys/af2_lir_screen.

## References

[1] Wild P, Farhan H, McEwan DG, et al. Phosphorylation of the autophagy receptor optineurin restricts *salmonella* growth. Science. 2011;333(6039):228–233. doi: 10.1126/science.1205405 21617041 PMC3714538

[2] Zhao D, Zou C-X, Liu X-M, et al. A UPR-Induced soluble ER-Phagy receptor acts with VAPs to confer ER stress resistance. Mol Cell. 2020;79(6):963–977.e3. doi: 10.1016/j.molcel.2020.07.019 32735772

[3] Liu L, Feng D, Chen G, et al. Mitochondrial outer-membrane protein FUNDC1 mediates hypoxia-induced mitophagy in mammalian cells. Nat Cell Biol. 2012;14(2):177–185. doi: 10.1038/ncb2422 22267086

[4] Wilfling F, Lee C-W, Erdmann PS, et al. A selective autophagy pathway for phase-separated endocytic protein deposits. Mol Cell. 2020;80(5):764–778.e7. doi: 10.1016/j.molcel.2020.10.030 33207182 PMC7721475

[5] Hyttinen JMT, Amadio M, Viiri J, et al. Clearance of misfolded and aggregated proteins by aggrephagy and implications for aggregation diseases. Ageing Res Rev. 2014;18:16–28. doi: 10.1016/j.arr.2014.07.002 25062811

[6] Dikic I, Elazar Z. Mechanism and medical implications of mammalian autophagy. Nat Rev Mol Cell Biol. 2018;19(6):349–364. doi: 10.1038/s41580-018-0003-4 29618831

[7] Lamark T, Johansen T. Mechanisms of selective autophagy. Annu Rev Cell Dev Biol. 2021;37(1):143–169. doi: 10.1146/annurev-cellbio-120219-035530 34152791

[8] Wu M-Y, Li Z-W, Lu J-H. Molecular modulators and receptors of selective autophagy: disease implication and identification strategies. Int J Biol Sci. 2024;20(2):751–764. doi: 10.7150/ijbs.83205 38169614 PMC10758101

[9] Rogov V, Dötsch V, Johansen T, et al. Interactions between autophagy receptors and ubiquitin-like proteins form the molecular basis for selective autophagy. Mol Cell. 2014;53(2):167–178. doi: 10.1016/j.molcel.2013.12.014 24462201

[10] Mizushima N. The ATG conjugation systems in autophagy. Curr Opin Cell Biol. 2020;63:1–10. doi: 10.1016/j.ceb.2019.12.001 31901645

[11] Lee C-W, Wilfling F, Ronchi P, et al. Selective autophagy degrades nuclear pore complexes. Nat Cell Biol. 2020;22(2):159–166. doi: 10.1038/s41556-019-0459-2 32029894

[12] Marshall RS, Hua Z, Mali S, et al. ATG8-binding UIM proteins define a new class of autophagy adaptors and receptors. Cell. 2019;177(3):766–781.e24. doi: 10.1016/j.cell.2019.02.009 30955882 PMC6810650

[13] Rogov N VV, Tsapras IP, Tsapras P, et al. Atg8 family proteins, LIR/AIM motifs and other interaction modes. Autophagy Rep. 2023;2(1):2188523. doi: 10.1080/27694127.2023.2188523 38214012 PMC7615515

[14] Chatzichristofi A, Sagris V, Pallaris A, et al. Lircentral: a manually curated online database of experimentally validated functional LIR motifs. Autophagy. 2023;19(12):3189–3200. doi: 10.1080/15548627.2023.2235851 37530436 PMC10621281

[15] Farnung J, Muhar M, Liang JR, et al. Semisynthetic LC3 probes for autophagy pathways reveal a noncanonical LC3 interacting region motif crucial for the enzymatic activity of human ATG3. ACS Cent Sci. 2023;9(5):1025–1034. doi: 10.1021/acscentsci.3c00009 37252361 PMC10214526

[16] Stephani M, Picchianti L, Gajic A, et al. A cross-kingdom conserved ER-phagy receptor maintains endoplasmic reticulum homeostasis during stress. Elife. 2020;9:e58396. doi: 10.7554/eLife.58396 32851973 PMC7515635

[17] von Muhlinen N, Akutsu M, Ravenhill BJ, et al. LC3C, bound selectively by a noncanonical LIR motif in NDP52, is required for antibacterial autophagy. Mol Cell. 2012;48(3):329–342. doi: 10.1016/j.molcel.2012.08.024 23022382 PMC3510444

[18] Shrestha BK, Skytte Rasmussen M, Abudu YP, et al. NIMA-related kinase 9–mediated phosphorylation of the microtubule-associated LC3B protein at thr-50 suppresses selective autophagy of p62/sequestosome 1. J Biol Chem. 2020;295(5):1240–1260. doi: 10.1016/S0021-9258(17)49883-8 31857374 PMC6996884

[19] Huber J, Obata M, Gruber J, et al. An atypical LIR motif within UBA5 (ubiquitin like modifier activating enzyme 5) interacts with GABARAP proteins and mediates membrane localization of UBA5. Autophagy. 2020;16(2):256–270. doi: 10.1080/15548627.2019.1606637 30990354 PMC6984602

[20] Keown JR, Black MM, Ferron A, et al. A helical LC3-interacting region mediates the interaction between the retroviral restriction factor Trim5α and mammalian autophagy-related ATG8 proteins. J Biol Chem. 2018;293(47):18378–18386. doi: 10.1074/jbc.RA118.004202 30282803 PMC6254359

[21] Ma P, Schwarten M, Schneider L, et al. Interaction of bcl-2 with the autophagy-related GABAA receptor-associated protein (GABARAP). J Biol Chem. 2013;288(52):37204–37215. doi: 10.1074/jbc.M113.528067 24240096 PMC3873574

[22] Kaufmann A, Beier V, Franquelim HG, et al. Molecular mechanism of autophagic membrane-scaffold assembly and disassembly. Cell. 2014;156(3):469–481. doi: 10.1016/j.cell.2013.12.022 24485455

[23] Weiergräber OH, Stangler T, Thielmann Y, et al. Ligand binding mode of GABAA receptor-associated protein. J Mol Biol. 2008;381(5):1320–1331. doi: 10.1016/j.jmb.2008.06.086 18638487

[24] Popelka H, Klionsky DJ. Analysis of the native conformation of the LIR/AIM motif in the Atg8/LC3/GABARAP-binding proteins. Autophagy. 2015;11(12):2153–2159. doi: 10.1080/15548627.2015.1111503 26565669 PMC4835208

[25] Rogov VV, Suzuki H, Fiskin E, et al. Structural basis for phosphorylation-triggered autophagic clearance of *Salmonella*. Biochemical J. 2013;454(3):459–466. doi: 10.1042/BJ20121907 23805866

[26] Rogov VV, Suzuki H, Marinković M, et al. Phosphorylation of the mitochondrial autophagy receptor Nix enhances its interaction with LC3 proteins. Sci Rep. 2017;7(1):1131. doi: 10.1038/s41598-017-01258-6 28442745 PMC5430633

[27] Wirth M, Zhang W, Razi M, et al. Molecular determinants regulating selective binding of autophagy adapters and receptors to ATG8 proteins. Nat Commun. 2019;10(1):2055. doi: 10.1038/s41467-019-10059-6 31053714 PMC6499816

[28] Kim R, Koh J. Biophysical characterization of the interaction of Atg8 with a disordered region of Nup159 involved in selective autophagy of the nuclear pore complex. Biochem Biophys Res Commun. 2022;604:172–178. doi: 10.1016/j.bbrc.2022.03.056 35306250

[29] Jumper J, Evans R, Pritzel A, et al. Highly accurate protein structure prediction with AlphaFold. Nature. 2021;596(7873):583–589. doi: 10.1038/s41586-021-03819-2 34265844 PMC8371605

[30] Kalvari I, Tsompanis S, Mulakkal NC, et al. iLIR: a web resource for prediction of Atg8-family interacting proteins. Autophagy. 2014;10(5):913–925. doi: 10.4161/auto.28260 24589857 PMC5119064

[31] Ibrahim T, Khandare V, Mirkin FG, et al. AlphaFold2-multimer guided high-accuracy prediction of typical and atypical ATG8-binding motifs. Simonsen A, editor. PLOS Biol. 2023;21(2):e3001962. doi: 10.1371/journal.pbio.3001962 36753519 PMC9907853

[32] Evans R, O’Neill M, Pritzel A, et al. Protein complex prediction with AlphaFold-Multimer. bioRxiv; 2022 [cited 2024 Apr 15]. Available from: https://www.biorxiv.org/content/10.1101/2021.10.04.463034v2

[33] Cristiani A, Dutta A, Poveda-Cuevas SA, et al. Identification of potential selective autophagy receptors from protein-content profiling of autophagosomes. J Cell Biochem. 2023. doi: 10.1002/jcb.30405 37087736

[34] Savinov A, Swanson S, Keating AE, et al. High-throughput computational discovery of inhibitory protein fragments with AlphaFold. bioRxiv; 2023 [cited 2024 Apr 15]. Available from: https://www.biorxiv.org/content/10.1101/2023.12.19.572389v1 10.1073/pnas.2322412122 PMC11831152 39899719

[35] Lee CY, Hubrich D, Varga JK, et al. Systematic discovery of protein interaction interfaces using AlphaFold and experimental validation. Mol Syst Biol. 2024;20(2):75–97. doi: 10.1038/s44320-023-00005-6 38225382 PMC10883280

[36] Cappadocia L, Lima CD. Ubiquitin-like protein conjugation: structures, chemistry, and mechanism. Chem Rev. 2018;118(3):889–918. doi: 10.1021/acs.chemrev.6b00737 28234446 PMC5815371

[37] Yau T-Y, Sander W, Eidson C, et al. SUMO interacting motifs: structure and function. Cells. 2021;10(11):2825. doi: 10.3390/cells10112825 34831049 PMC8616421

[38] Lascorz J, Codina-Fabra J, Reverter D, et al. SUMO-SIM interactions: from structure to biological functions. Semin Cell Dev Biol. 2022;132:193–202. doi: 10.1016/j.semcdb.2021.11.007 34840078

[39] Celen AB, Sahin U. Sumoylation on its 25th anniversary: mechanisms, pathology, and emerging concepts. FEBS J. 2020;287(15):3110–3140. doi: 10.1111/febs.15319 32255256

[40] Zhao Q, Xie Y, Zheng Y, et al. GPS-SUMO: a tool for the prediction of sumoylation sites and SUMO-interaction motifs. Nucleic Acids Res. 2014;42(W1):W325–W330. doi: 10.1093/nar/gku383 24880689 PMC4086084

[41] Beauclair G, Bridier-Nahmias A, Zagury J-F, et al. JASSA: a comprehensive tool for prediction of SUMOylation sites and SIMs. Bioinformatics. 2015;31(21):3483–3491. doi: 10.1093/bioinformatics/btv403 26142185

[42] Gou Y, Liu D, Chen M, et al. GPS-SUMO 2.0: an updated online service for the prediction of SUMOylation sites and SUMO-interacting motifs. Nucleic Acids Res. 2024;52(W1):W238–W247. doi: 10.1093/nar/gkae346 38709873 PMC11223847

[43] Abramson J, Adler J, Dunger J, et al. Accurate structure prediction of biomolecular interactions with AlphaFold 3. Nature. 2024;630(8016):493–500. doi: 10.1038/s41586-024-07487-w 38718835 PMC11168924

[44] Zientara-Rytter K, Łukomska J, Moniuszko G, et al. Identification and functional analysis of Joka2, a tobacco member of the family of selective autophagy cargo receptors. Autophagy. 2011;7(10):1145–1158. doi: 10.4161/auto.7.10.16617 21670587 PMC3242614

[45] Mohrlüder J, Stangler T, Hoffmann Y, et al. Identification of calreticulin as a ligand of GABARAP by phage display screening of a peptide library. FEBS J. 2007;274(21):5543–5555. doi: 10.1111/j.1742-4658.2007.06073.x 17916189

[46] Thielmann Y, Weiergräber OH, Mohrlüder J, et al. Structural framework of the GABARAP–calreticulin interface – implications for substrate binding to endoplasmic reticulum chaperones. FEBS J. 2009;276(4):1140–1152. doi: 10.1111/j.1742-4658.2008.06857.x 19154346

[47] Li Z, Li S, Luo M, et al. dbPTM in 2022: an updated database for exploring regulatory networks and functional associations of protein post-translational modifications. Nucleic Acids Res. 2022;50(D1):D471–D479. doi: 10.1093/nar/gkab1017 34788852 PMC8728263

[48] Sanner MF, Olson AJ, Spehner JC. Reduced surface: an efficient way to compute molecular surfaces. Biopolymers. 1996;38(3):305–320. doi: 10.1002/(SICI)1097-0282(199603)38:3<305::AID-BIP4>3.0.CO;2-Y 8906967

[49] Cock PJA, Antao T, Chang JT, et al. Biopython: freely available python tools for computational molecular biology and bioinformatics. Bioinformatics. 2009;25(11):1422–1423. doi: 10.1093/bioinformatics/btp163 19304878 PMC2682512

[50] Jiang S, Wells CD, Roach PJ. Starch-binding domain-containing protein 1 (Stbd1) and glycogen metabolism: identification of the Atg8 family interacting motif (AIM) in Stbd1 required for interaction with GABARAPL1. Biochem Biophys Res Commun. 2011;413(3):420–425. doi: 10.1016/j.bbrc.2011.08.106 21893048 PMC3411280

[51] Zhu Y, Massen S, Terenzio M, et al. Modulation of serines 17 and 24 in the LC3-interacting region of Bnip3 determines pro-survival mitophagy versus apoptosis. J Biol Chem. 2013;288(2):1099–1113. doi: 10.1074/jbc.M112.399345 23209295 PMC3542995

[52] Hanna RA, Quinsay MN, Orogo AM, et al. Microtubule-associated protein 1 light chain 3 (LC3) interacts with Bnip3 protein to selectively remove endoplasmic reticulum and mitochondria via autophagy. J Biol Chem. 2012;287(23):19094–19104. doi: 10.1074/jbc.M111.322933 22505714 PMC3365942

[53] Alemu EA, Lamark T, Torgersen KM, et al. ATG8 family proteins act as scaffolds for assembly of the ULK complex. J Biol Chem. 2012;287(47):39275–39290. doi: 10.1074/jbc.M112.378109 23043107 PMC3501051

[54] Nolan TM, Brennan B, Yang M, et al. Selective autophagy of BES1 mediated by DSK2 balances plant growth and survival. Dev Cell. 2017;41(1):33–46.e7. doi: 10.1016/j.devcel.2017.03.013 28399398 PMC5720862

[55] Kimura T, Jain A, Choi SW, et al. TRIM-mediated precision autophagy targets cytoplasmic regulators of innate immunity. J Cell Biol. 2015;210(6):973–989. doi: 10.1083/jcb.201503023 26347139 PMC4576868

[56] Carroll B, Mohd-Naim N, Maximiano F, et al. The TBC/RabGAP Armus coordinates Rac1 and Rab7 functions during autophagy. Dev Cell. 2013;25(1):15–28. doi: 10.1016/j.devcel.2013.03.005 23562278 PMC3898768

[57] Chino H, Yamasaki A, Ode KL, et al. Phosphorylation by casein kinase 2 enhances the interaction between ER‐phagy receptor TEX264 and ATG8 proteins. EMBO Rep. 2022;23(6):e54801. doi: 10.15252/embr.202254801 35417087 PMC9171416

[58] Rogov VV, Stolz A, Ravichandran AC, et al. Structural and functional analysis of the GABARAP interaction motif (GIM). EMBO Rep. 2017;18(8):1382–1396. doi: 10.15252/embr.201643587 28655748 PMC5538626

[59] McEwan DG, Popovic D, Gubas A, et al. PLEKHM1 regulates autophagosome-lysosome fusion through HOPS complex and LC3/GABARAP proteins. Mol Cell. 2015;57(1):39–54. doi: 10.1016/j.molcel.2014.11.006 25498145

[60] Kuang Y, Ma K, Zhou C, et al. Structural basis for the phosphorylation of FUNDC1 LIR as a molecular switch of mitophagy. Autophagy. 2016;12(12):2363–2373. doi: 10.1080/15548627.2016.1238552 27653272 PMC5173264

[61] Beck M, Hurt E. The nuclear pore complex: understanding its function through structural insight. Nat Rev Mol Cell Biol. 2017;18(2):73–89. doi: 10.1038/nrm.2016.147 27999437

[62] Wesch N, Kirkin V, Rogov VV., et al. Atg8-family proteins—structural features and molecular interactions in autophagy and beyond. Cells. 2020;9(9):2008. doi: 10.3390/cells9092008 32882854 PMC7564214

[63] Cappadocia L, Mascle XH, Bourdeau V, et al. Structural and functional characterization of the phosphorylation-dependent interaction between PML and SUMO1. Structure. 2015;23(1):126–138. doi: 10.1016/j.str.2014.10.015 25497731

[64] Anamika A, Spyracopoulos L. Molecular basis for phosphorylation-dependent SUMO recognition by the DNA repair protein RAP80*. J Biol Chem. 2016;291(9):4417–4428. doi: 10.1074/jbc.M115.705061 26719330 PMC4813470

[65] Berman HM. The protein data bank. Nucleic Acids Res. 2000;28(1):235–242. doi: 10.1093/nar/28.1.235 10592235 PMC102472

[66] Riboldi GM, Faravelli I, Kuwajima T, et al. Sumoylation regulates the assembly and activity of the SMN complex. Nat Commun. 2021;12(1):5040. doi: 10.1038/s41467-021-25272-5 34413305 PMC8376998

[67] Diao X, Guo C, Zheng H, et al. Sumoylation-triggered ALIX activation modulates extracellular vesicles circTLCD4-RWDD3 to promote lymphatic metastasis of non-small cell lung cancer. Sig Transduct Target Ther. 2023;8(1):426. doi: 10.1038/s41392-023-01685-0 PMC10625632 37925421

[68] Ivarsson Y, Jemth P. Affinity and specificity of motif-based protein–protein interactions. Curr Opin Struct Biol. 2019;54:26–33. doi: 10.1016/j.sbi.2018.09.009 30368054

[69] Banani SF, Rice AM, Peeples WB, et al. Compositional control of phase-separated cellular bodies. Cell. 2016;166(3):651–663. doi: 10.1016/j.cell.2016.06.010 27374333 PMC4967043

[70] Kötter A, Mootz HD, Heuer A. Conformational and interface variability in multivalent SIM–SUMO interaction. J Phys Chem B. 2023;127(17):3806–3815. doi: 10.1021/acs.jpcb.2c08760 37079893

[71] Johansen T, Lamark T. Selective autophagy: ATG8 family proteins, LIR motifs and cargo receptors. J Mol Biol. 2020;432(1):80–103. doi: 10.1016/j.jmb.2019.07.016 31310766

[72] Antonescu ON, Utichi M, Sora V, et al. Decoding phospho-regulation and flanking regions in autophagy-associated short linear motifs: a case study of optineurin-LC3B interaction. bioRxiv; 2023 [cited 2024 Apr 15]. Available from: https://www.biorxiv.org/content/10.1101/2023.09.30.560296v2 10.1038/s42003-025-08399-9 PMC12368060 40835742

[73] Mascle XH, Gagnon C, Wahba HM, et al. Acetylation of SUMO1 alters interactions with the SIMs of PML and Daxx in a protein-specific manner. Structure. 2020;28(2):157–168.e5. doi: 10.1016/j.str.2019.11.019 31879127

[74] Glukhov E, Averkava V, Kotelnikov S, et al. Phospho-tune: enhanced structural modeling of phosphorylated protein interactions. bioRxiv; 2024 [cited 2024 Apr 15. Available from: https://www.biorxiv.org/content/10.1101/2024.02.29.582580v1

[75] Ahdritz G, Bouatta N, Floristean C, et al. OpenFold: retraining AlphaFold2 yields new insights into its learning mechanisms and capacity for generalization. Nat Methods. 2024;21(8):1514–1524. doi: 10.1038/s41592-024-02272-z 38744917 PMC11645889

[76] Baek M, Anishchenko I, Humphreys IR, et al. Efficient and accurate prediction of protein structure using RoseTTAFold2. bioRxiv; 2023 [cited 2024 Jul 31]. Available from: https://www.biorxiv.org/content/10.1101/2023.05.24.542179v1

[77] Bateman A, Martin M-J, Orchard S. The UniProt consortium. UniProt: the universal protein knowledgebase in 2023. Nucleic Acids Res. 2023;51(D1):D523–D531. doi: 10.1093/nar/gkac1052 36408920 PMC9825514

[78] Yu D, Chojnowski G, Rosenthal M, et al. AlphaPulldown—a python package for protein–protein interaction screens using AlphaFold-multimer. Cowen L, editor. Bioinformatics. 2023;39(1):btac749. doi: 10.1093/bioinformatics/btac749 36413069 PMC9805587

[79] Mirdita M, Schütze K, Moriwaki Y, et al. ColabFold: making protein folding accessible to all. Nat Methods. 2022;19(6):679–682. doi: 10.1038/s41592-022-01488-1 35637307 PMC9184281

[80] Picchianti L, Sánchez De Medina Hernández V, Zhan N, et al. Shuffled ATG8 interacting motifs form an ancestral bridge between UFMylation and autophagy. Embo J. 2023;42(10):e112053. doi: 10.15252/embj.2022112053 36762703 PMC10183829

[81] Kabsch W, Sander C. Dictionary of protein secondary structure: pattern recognition of hydrogen‐bonded and geometrical features. Biopolymers. 1983;22(12):2577–2637. doi: 10.1002/bip.360221211 6667333

[82] Schrödinger LLC. The PyMOL molecular graphics system, version 2.5.7. 2023.

[83] Abraham MJ, Murtola T, Schulz R, et al. GROMACS: high performance molecular simulations through multi-level parallelism from laptops to supercomputers. SoftwareX. 2015;1–2:19–25. doi: 10.1016/j.softx.2015.06.001

[84] Huang J, Rauscher S, Nawrocki G, et al. CHARMM36m: an improved force field for folded and intrinsically disordered proteins. Nat Methods. 2017;14(1):71–73. doi: 10.1038/nmeth.4067 27819658 PMC5199616

[85] Ruff KM, Pappu RV. AlphaFold and implications for intrinsically disordered proteins. J Mol Biol. 2021;433(20):167208. doi: 10.1016/j.jmb.2021.167208 34418423

[86] Varadi M, Bertoni D, Magana P, et al. AlphaFold protein structure database in 2024: providing structure coverage for over 214 million protein sequences. Nucleic Acids Res. 2024;52(D1):D368–D375. doi: 10.1093/nar/gkad1011 37933859 PMC10767828

[87] Berendsen HJC, Postma JPM, van Gunsteren WF, et al. Molecular dynamics with coupling to an external bath. J Chem Phys. 1984;81(8):3684–3690. doi: 10.1063/1.448118

[88] Bussi G, Donadio D, Parrinello M. Canonical sampling through velocity rescaling. J Chem Phys. 2007;126(1):014101. doi: 10.1063/1.2408420 17212484

[89] Parrinello M, Rahman A. Polymorphic transitions in single crystals: a new molecular dynamics method. J Appl Phys. 1981;52(12):7182–7190. doi: 10.1063/1.328693

[90] Páll S, Hess B. A flexible algorithm for calculating pair interactions on SIMD architectures. Comput Phys Commun. 2013;184(12):2641–2650. doi: 10.1016/j.cpc.2013.06.003

[91] Essmann U, Perera L, Berkowitz ML, et al. A smooth particle mesh Ewald method. J Chem Phys. 1995;103(19):8577–8593. doi: 10.1063/1.470117

[92] Hess B, Bekker H, Berendsen HJC, et al. LINCS: a linear constraint solver for molecular simulations. J Comput Chem. 1997;18(12):1463–1472. doi: 10.1002/(SICI)1096-987X(199709)18:12<1463::AID-JCC4>3.0.CO;2-H

[93] Humphrey W, Dalke A, Schulten K. VMD: visual molecular dynamics. J Mol Graphics. 1996;14(1):33–38. doi: 10.1016/0263-7855(96)00018-5 8744570

[94] Python {release} {python} 3.10.9. Python.org [Internet].

[95] Anaconda Software Distribution. [Internet]. 2020. Available from: https://docs.anaconda.com/

[96] Perez F, Granger BE. Ipython: a system for interactive scientific computing. Comput Sci Eng. 2007;9(3):21–29. doi: 10.1109/MCSE.2007.53

[97] Harris CR, Millman KJ, van der Walt SJ, et al. Array programming with NumPy. Nature. 2020;585(7825):357–362. doi: 10.1038/s41586-020-2649-2 32939066 PMC7759461

[98] Hunter JD. Matplotlib: a 2D graphics environment. Comput Sci Eng. 2007;9(3):90–95. doi: 10.1109/MCSE.2007.55

[99] Waskom M. Seaborn: statistical data visualization. JOSS. 2021;6(60):3021. doi: 10.21105/joss.03021

[100] Michaud-Agrawal N, Denning EJ, Woolf TB, et al. Mdanalysis: a toolkit for the analysis of molecular dynamics simulations. J Comput Chem. 2011;32(10):2319–2327. doi: 10.1002/jcc.21787 21500218 PMC3144279

[101] McGibbon RT, Beauchamp KA, Harrigan MP, et al. Mdtraj: a modern open library for the analysis of molecular dynamics trajectories. Biophys J. 2015;109(8):1528–1532. doi: 10.1016/j.bpj.2015.08.015 26488642 PMC4623899

