# Supplementary material for: AlphaFold2 SLiM screen for LC3-LIR interactions in autophagy
Source: Autophagy. 2025 May 4;21(10):2192–212. doi: 10.1080/15548627.2025.2493999 (PMC12582063; doi:10.1080/15548627.2025.2493999)
Supplement: SI_Stuke_R3.docx [file KAUP_A_2493999_SM4766.docx]

Supplementary Information:

AlphaFold2 SLiM screen for LC3-LIR interactions in autophagy

Jan Felix Maximilian Stuke^†^ and Gerhard Hummer^∗,†,‡^

†*Department of Theoretical Biophysics, Max Planck Institute of Biophysics, Max-von-Laue*

*Straße 3, 60438, Frankfurt am Main, Germany*

‡*Institute of Biophysics, Goethe University Frankfurt, Max-von-Laue Straße 9, 60438,*

*Frankfurt am Main, Germany*

*E-mail: gerhard.hummer@biophys.mpg.de

# Supplementary Figures

**Figure S1.** AlphaFold2.3 Multimer and AlphaFold3 increase the number of LC3-LIR interactions identified in predictions with full-length target proteins. (**A**) Atg8-Nup159 interaction is captured by AlphaFold2.3. Shown are 25 models of an AlphaFold2.3 prediction of the interaction between Atg8 (surface representation) and Nup159 (cartoon representation, with LIR residues Y1078 and L1081 highlighted as orange licorice), aligned on Atg8. Only the four core AIM1_1078-1081_ residues (1078-1081) of Nup159 from the respective model, and only Atg8 from the top model are shown for clarity. AlphaFold2.2 did not predict this interaction in 25 models (data not shown). (**B**) ATG8CL-Joka2 interaction is suggested by AlphaFold3. Shown are 25 models of an AlphaFold3 prediction of the interaction between ATG8CL (surface representation) and Joka2 (cartoon representation, with LIR residues W821 and I824 highlighted as orange licorice), structurally aligned on ATG8CL. Only the four core LIR_821-824_ residues (821-824) of Joka2 from the respective model and only ATG8CL from the top model are shown for clarity. AlphaFold2.2 and 2.3 did not predict this interaction in 25 models (data not shown). (**C and D**) AlphaFold2.3 does not capture the LC3-LIR interaction of GABARAP with full-length CALR (calreticulin). In **C**, shown are 25 models of an AlphaFold2.3 prediction of the interaction between GABARAP (surface representation) and CALR (cartoon representation, with LIR residues W200 and L203 highlighted as orange licorice), aligned on CALR. In **D**, shown are the same 25 models as in **C**, aligned on GABARAP instead. Only the four core LIR_200-203_ residues (200-203) of CALR from the respective model and only GABARAP from the top model are shown for clarity. AlphaFold2.2 also did not predict this interaction in 25 models (data not shown). (**E**) GABARAP-CALR interaction is not captured by AlphaFold3. Shown are 25 models of an AlphaFold3 prediction of the interaction between GABARAP (surface representation) and CALR (cartoon representation, with LIR residues W200 and L203 highlighted as orange licorice), structurally aligned on GABARAP. Only the four core LIR_200-203_ residues (200-203) of CALR from the respective model, and only GABARAP from the top model are shown for clarity. All structures are colored by predicted local-distance difference test (pLDDT) score.

**Figure S2.** Fragment length and phosphomimetic mutations can modulate the prediction of LC3-LIR interactions with AlphaFold2, which can be exploited in systematic screens. Predicted local-distance difference test (pLDDT) score, minimum predicted aligned error (minPAE), and binding mode for the four LIR/AIM residues in AlphaFold2.2 predictions of (**A**) LC3B and OPTN fragments containing the LIR_178−181_ and (**B**) Atg8 and Nup159 fragments containing the AIM1_1078−1081_. Fragments were varied (i) in length and (ii) by the introduction of phosphomimetic S/T to E mutations for experimentally confirmed phosphosites. The dotted lines indicate the minimum length required for the multiple sequence alignment (MSA) of AlphaFold2. Stripes above the pLDDT curve/below the minPAE curve indicate the binding mode of the respective fragment with phosphomimetic mutations (green: canonical LIR; yellow: ncLIR; light blue: lcLIR; gray: other). Stripes below the pLDDT curve/above the minPAE curve indicate the binding mode of the respective wild-type fragment. (**C**) Overview over the LIR scanning pipeline. Inputs are sequences of the LC3 protein to be screened against and the wild-type (WT) target, optionally a target sequence with phosphomimetic (ST) mutations, and a structure of the target protein (all input files are marked with an asterisk). Overlapping fragments of WT and ST target sequences are then screened against the LC3 via AlphaPulldown [78]. The predicted complex structures are analyzed for LC3-LIR and other interactions. The input structure file provides information on the accessibility of the predicted interacting motifs in the target. Different fragment lengths provide a trade-off between sensitivity and specificity. The screen can be repeated for different fragment lengths and LC3s. The predicted binding motifs are grouped based on their similarity in a summary analysis. Relevant file formats are indicated for every step.

**Figure S3.** Fragment length modulates LC3-LIR complex predictions in the context of a systematic screen. Systematic screen of the interaction between 16- and 52-residue fragments of (**A**) OPTN screened against LC3B, (**B**) CALR screened against GABARAP, and (**C**) Nup159 against Atg8, respectively. The fragments have a 75% overlap. We performed the screen for the wild-type (WT) sequence, and a phosphomimetic (ST) sequence, in which S/T to E mutations were introduced at experimentally confirmed phosphosites. Predicted local-distance difference test (pLDDT) score, minimum predicted aligned error (minPAE), and binding mode for the interacting residues are shown (minimum minPAE from all fragments covering each residue and its respective pLDDT score). Stripes above the pLDDT curve/below the minPAE curve indicate the binding mode of the respective fragment with phosphomimetic mutations (see legend). Stripes below the pLDDT curve/above the minPAE curve indicate the binding mode of the respective wild-type fragment. Additionally, we calculated residue depth (r.d.) and secondary structure (sec. str.) from the AlphaFold2-predicted structure for the full-length protein (only for residues with pLDDT $\geq$ 70 in the full-length structure) to add structural context to the sequence. The experimentally confirmed LIRs are indicated at the top by orange triangles. (**D**) Results of fragment-prediction screens for Nup159 combined with Atg8. Shown are the top fifteen predicted LIRs, sorted by relative occurrence in longer fragments (52mers *>* 36mers *>* 16mers). Residues binding in hydrophobic pocket 0, 1, and 2 are highlighted in bold. The type of interaction is indicated below each motif. The experimentally confirmed LIR is underlined in orange, the experimentally non-functional LIRs in purple.

**Figure S4.** Functional and non-functional LIRs are not as readily distinguished by their top AlphaFold2.3 scores than by their length-weighted fraction of occurrence (LO) score shown in Fig. 4. (**A**) Cumulative distribution functions (CDFs) of the best core-motif pLDDT score (averaged over the 4 highest scoring consecutive residues in the respective fragment) in any fragment for all predicted and expected motifs from our benchmark data set (detailed data shown in Fig. 3 and Fig. 5) grouped by experimentally confirmed functional motifs (in orange), experimentally confirmed non-functional motifs (purple), and motifs with unknown functionality (gray). Solid lines show the scores for wild-type (WT) and dotted lines the scores for phosphomimetic (ST) predictions. Experimentally confirmed or non-confirmed motifs that were not found in the predictions were scored with 0. (**B**) Cumulative distribution functions of the best core-motif minPAE score (averaged over the 4 lowest scoring consecutive residues in the respective fragment) in any fragment for the same data set as in **A.** Experimentally confirmed or non-confirmed motifs that were not found in the predictions were scored with 8 Å. (**C**) Probability distribution function (PDF) for the distance (in residues) between the closest impactful (either changes binding mode or moves the fragment over/below the detection cutoff) phosphomimetic mutation and the respective interaction motif. A distance of 0 indicates that the mutation resides within the motif. Data from all screens of the benchmark set. (**D, E, and F**) Results of fragment-prediction screens for (**D**) LC3B and OPTN, (**E**) GABARAP and CALR, (**F**) Atg8 and Nup159. Shown are the top five (15 for Atg8 and Nup159) predicted LIRs with known interaction type and an average residue depth ≤ 0.3 sorted by LO score (averaged over WT and ST runs). Residues binding in hydrophobic pocket 0, 1, and 2 are highlighted in bold. The type of interaction is indicated below each motif. The experimentally confirmed functional LIRs are underlined in orange, the experimentally confirmed non-functional LIRs in purple.

**Figure S5.** Detailed analysis of full-length and fragment structures predicted by AlphaFold2 Multimer assists in the interpretation of screen results. (**A**) Structural occlusion of the predicted LIR_637−640_ in Joka2 shown in the cartoon representation (W637 and L640 highlighted as orange licorice) of the full-length AlphaFold2 model (only the relevant domain is shown for visual clarity) and via calculated residue depth (r.d.) and secondary structure (sec. str.). Both properties were calculated from the full-length structure for residues with pLDDT $\geq$ 70 in that structure. The predicted LIR_637−640_ is indicated via a hollow orange triangle. (**B**) Structural occlusion of the experimentally confirmed functional LIR_43−46_ in DSK2A shown in the cartoon representation (F43 and L46 highlighted as orange licorice) of the full-length AlphaFold2 model (only the relevant domain is shown for visual clarity). (**C**) Representative AlphaFold2 models of DSK2A fragments (cartoon representation) containing LIR_43−46_ (F43 and L46 highlighted as orange licorice) and interacting with ATG8E (surface representation) in different interaction modes. (**D**) Representative AlphaFold2 models of MEFV fragments (cartoon representation) (partially) containing LIR_470−477_ (F472 and L473 highlighted as orange licorice) and interacting with GABARAP (surface representation) in different interaction modes. Structures in **A**, **B**, **C,** and **D** are colored by predicted local-distance difference test (pLDDT) score. (**E**) Systematic screen of the interaction between 36-residue fragments of C53 (*A.t.*) with ATG8E and CDK5RAP3 (*H*.*s.*) with GABARAP, respectively. The fragments have a 75% overlap. We performed the screen for the wild-type (WT) sequence and a phosphomimetic (ST) sequence, in which S/T to E mutations were introduced at experimentally confirmed phosphosites. pLDDT score, minimum predicted aligned error (minPAE), and binding mode for the interacting residues are shown (minimum minPAE from all fragments covering each residue and its respective pLDDT score). Stripes above the pLDDT curve/below the minPAE curve indicate the binding mode of the respective fragment with phosphomimetic mutations. Stripes below the pLDDT curve/above the minPAE curve indicate the binding mode of the respective wild-type fragment. Additionally, we calculated residue depth (r.d.) and secondary structure (sec. str.) from the AlphaFold2-predicted structure for the full-length protein (only for residues with pLDDT $\geq$ 70 in the full-length structure) to add structural context to the sequence. The experimentally confirmed LIRs are indicated at the top by filled orange triangles, the predicted LIR_320−324_ as a hollow orange triangle. (**F**) Results of fragment prediction screens for C53 (*A.t.*) combined with ATG8E. Shown are the top fifteen predicted LIRs with known interaction types, sorted by LO score. Predicted motifs with an average residue depth $\geq$ 0.3 nm are not shown. Residues binding in hydrophobic pocket 0, 1, and 2 are highlighted in bold. The type of interaction is indicated below the motif. The experimentally confirmed LIR is underlined in orange, the experimentally non-functional LIRs (in different binding modes) in purple.

**Figure S6.** Nup159 AIM1 secondary structure in MD simulations. (**A**) Previously determined crystal structure of the TEX264 LIR_273-276_ with a phosphomimetic mutation (S272D) bound to GABARAP (PDB ID: 7VED) [102]. D272 residue from TEX264 and interacting residues from GABARAP are highlighted as licorice. (**B**) Plots show the secondary structure (colors as indicated in legend) of a 20-residue Nup159 fragment containing AIM1_1078−1081_ over time. Data from triplicate (1 µs each, columns 1-3) MD simulations of the respective fragment in complex with Atg8 for five different systems (rows): unphosphorylated (SER/THR), unphosphorylated starting from the predicted structure with phosphomimetics (SER/THR-helix) phosphomimetic mutation (GLU), and phosphorylated (SP1/TP1: -HPO_4_^−^ and SP2/TP2: -PO_4_^2-^, respectively).

**Figure S7.** Sequence fragment scans for PLEKHM1, ULK1, and FUNDC1 with LC3B or GABARAP. Systematic screen of the interaction between 36-residue fragments of (**A**) PLEKHM1, (**B**) ULK1, and (**C**) FUNDC1 with LC3B and GABARAP, respectively. The fragments have 75% overlap with the adjacent fragments. We performed the screen for the wild-type (WT) sequence, and a phosphomimetic (ST) sequence, in which S/T to E mutations were introduced at experimentally confirmed phosphosites. Predicted local-distance difference test (pLDDT) score, minimum predicted aligned error (minPAE), and binding mode for the interacting residues are shown (minimum minPAE from all fragments covering each residue and its respective pLDDT score). Stripes above the pLDDT curve/below the minPAE curve indicate the binding mode of the respective fragment with phosphomimetic mutations. Stripes below the pLDDT curve/above the minPAE curve indicate the binding mode of the respective wild-type fragment. Additionally, we calculated residue depth (r.d.) and secondary structure (sec. str.) from the AlphaFold2-predicted structure for the full-length protein (only for residues with pLDDT $\geq$ 70 in the full-length structure) to add structural context to the sequence. The experimentally confirmed LIRs are indicated at the top by orange triangles.

**Figure S8.** Phosphomimetic mutations and phosphorylations alter the interactions of predicted LIRs in NUP214 with LC3B and GABARAP. (**A**) Systematic screen of the interaction between 36-residue fragments of NUP214 with LC3B and GABARAP, respectively. The fragments have 75% overlap. We performed the screen for the wild-type (WT) sequence, and a phosphomimetic (ST) sequence, in which S/T to E mutations were introduced at experimentally confirmed phosphosites. Predicted local-distance difference test (pLDDT) score, minimum predicted aligned error (minPAE), and binding mode for the interacting residues are shown (minimum minPAE from all fragments covering each residue and its respective pLDDT score). Stripes above the pLDDT curve/below the minPAE curve indicate the binding mode of the respective fragment with phosphomimetic mutations. Stripes below the pLDDT curve/above the minPAE curve indicate the binding mode of the respective wildtype fragment. Additionally, we calculated residue depth (r.d.) and secondary structure (sec. str.) from the AlphaFold2-predicted structure for the full-length protein (only for residues with pLDDT $\geq$ 70 in the full-length structure) to add structural context to the sequence. (**B and C**) Average number of heavy atom contacts (Av. num. of contacts; distance lower than 5 Å) per frame between potentially phosphorylated NUP214 residues and (**B**) LC3B and (**C**) GABARAP residues. Data from triplicate (1 *µ*s each) MD simulations for four different phosphorylation states: unphosphorylated (SER), phosphomimetic mutation (GLU), and phosphorylated (SP1: HPO_4_^−^ and SP2: -PO_4_^2-^, respectively). (**D**) Final snapshots from 1 µs MD simulations of LC3B with a NUP214 fragment containing LIR_713−720_ in four different phosphorylation states (3 replicas per state): unphosphorylated (light blue), phosphomimetic mutation (yellow), and phosphorylated (TP1: -HPO_4_^−^ and TP2: -PO_4_^2-^, red and orange respectively). All structures are aligned on LC3B. LC3B and the NUP214 fragment are shown in cartoon representation.

**Figure S9.** Sequence fragment scans for UIMC1, SMN1, and PDCD6IP with SUMO2. Systematic screen of the interaction between 36-residue fragments of (**A**) UIMC1, (**B**) SMN1, and (**C**) PDCD6IP with SUMO2. The fragments have 75% overlap. We performed the screen for the wild-type (WT) sequence, and a phosphomimetic (ST) sequence, in which S/T to E mutations were introduced at experimentally confirmed phosphosites. Predicted local-distance difference test (pLDDT) score, minimum predicted aligned error (minPAE), and binding mode for the interacting residues are shown (minimum minPAE from all fragments covering each residue and its respective pLDDT score). Stripes above the pLDDT curve/below the minPAE curve indicate the binding mode of the respective fragment with phosphomimetic mutations (see legend). Stripes below the pLDDT curve/above the minPAE curve indicate the binding mode of the respective wild-type fragment. Additionally, we calculated residue depth (r.d.) and secondary structure (sec. str.) from the AlphaFold2-predicted structure for the full-length protein (only for residues with pLDDT $\geq$ 70 in the full-length structure) to add structural context to the sequence. The experimentally confirmed SIMs are indicated at the top by orange triangles. (**D**) Summary of the results of a fragment-prediction screen for SMN1 combined with SUMO2. Shown are the top 5 predicted interacting motifs, sorted by relative occurrence in longer fragments (52mers > 36mers >16mers). Interaction types are indicated below the motif. The experimentally identified SIM is underlined in orange. (**E**) AlphaFold2.3 prediction for the interaction between SUMO2 (cartoon representation with transparent surface) and a 52-residue SMN1 fragment containing the experimentally confirmed SIM_124-127_ (cartoon representation with SIM residues highlighted as orange licorice). In the model, the SIM residues form the core of a small, folded domain that interacts with SUMO2 at a different site than the SIM-binding site.

# Supplementary Tables

**Table S1.** Overview over experimentally confirmed LIRs and SIMs for the proteins investigated with the fragment screen.

| **Name** | Joka2 | AT5G06830/  C53 (*A.t*.) | Nup159 | DSK2A | BNIP3 | CALR | CDK5RAP3/  C53 (*H.s*.) | STBD1 | ULK2 | OPTN |
| --- | --- | --- | --- | --- | --- | --- | --- | --- | --- | --- |
| **Confirmed LIRs/SIMs w/ str.** |  |  |  |  |  | 200-WDFL-203 |  |  |  | 178-FVEI-181 |
| **Confirmed LIRs/SIMs w/o str.** | 821-WDPI-824 | 304-YEIV-307 | 1078-YDKL-1081 | 43-FKEL-46 | 18-WVEL-21 |  | 267-IDWG-270 | 203-WEMV-206 | 353-FVLV-356 |  |
|  |  | 274-IDWD-277 |  | 249-FNML-252 |  |  | 292-IDWG-295 |  |  |  |
|  |  | 285-IDWD-288 |  | 256-YENV-259 |  |  | 310-IDWG-313 |  |  |  |
|  |  | 333-ISWD-336 |  |  |  |  |  |  |  |  |
| **Confirmed non-LIRs/SIMs** |  | 48-FSSL-51 | 1218-FQTV-1221 |  |  |  |  |  |  |  |
|  |  | 69-YLEV-72 | 1225-FTVL-1228 |  |  |  |  |  |  |  |
|  |  | 76-YEIL-79 | 1317-YLFL-1320 |  |  |  |  |  |  |  |
|  |  | 100-WEAI-103 | 1434-FKVV-1437 |  |  |  |  |  |  |  |
| **Reference** | <https://doi.org/10.4161/auto.7.10.16617> | <https://doi.org/10.7554/elife.58396> | <https://doi.org/10.1038/s41556-019-0459-2> | <https://doi.org/10.1016/j.devcel.2017.03.013> | <https://doi.org/10.1074/jbc.m111.322933> | <https://doi.org/10.1111/j.1742-4658.2008.06857.x> | <https://doi.org/10.7554/elife.58396> | <https://doi.org/10.1016/j.bbrc.2011.08.106> | <https://doi.org/10.1074/jbc.M112.378109> | <https://doi.org/10.1042/bj20121907> |
|  |  |  |  |  | <https://doi.org/10.1074/jbc.m112.399345> |  |  |  |  |  |
|  |  |  |  |  |  |  |  |  |  |  |
|  |  |  |  |  |  |  |  |  |  |  |
|  |  |  |  |  |  |  |  |  |  |  |
| **Name** | FUNDC1 | NUP214 | PLEKHM1 | ULK1 | MEFV/pyrin | TBC1D2/TBC1D2A | PDCD6IP/ALIX | UIMC1/RAP80 | SMN1/SMN |  |
| **Confirmed LIRs/SIMs w/ str.** | 353-FVLV-356 | - | 635-WVNV-638 | 357-FVMV-360 |  |  |  | 40-FIVISDSD-47 |  |  |
| **Confirmed LIRs/SIMs w/o str.** |  | - |  |  | 525-WELL-528 | 510-YLAGL-514 | 548-VVNV-551 |  | 124-VVVYTG-129 |  |
|  |  |  |  |  | 397-ICSLSHQEH-404 | 142-WEFHN-146 |  |  |  |  |
|  |  |  |  |  | 470-YYFLEQQE-477 |  |  |  |  |  |
|  |  |  |  |  |  |  |  |  |  |  |
| **Confirmed non-LIRs/SIMs** |  | - |  |  |  |  |  |  |  |  |
|  |  |  |  |  |  |  |  |  |  |  |
|  |  |  |  |  |  |  |  |  |  |  |
|  |  |  |  |  |  |  |  |  |  |  |
| **Reference** | <https://doi.org/10.1080/15548627.2016.1238552> |  | <https://doi.org/10.15252%2Fembr.201643587> | <https://doi.org/10.1074/jbc.m112.378109> | <https://doi.org/10.1083/jcb.201503023> | <https://doi.org/10.1016/j.devcel.2013.03.005> | <https://doi.org/10.1038/s41392-023-01685-0> | <https://doi.org/10.1074/jbc.M115.705061> | <https://doi.org/10.1038/s41467-021-25272-5> |  |
|  |  |  |  | <https://doi.org/10.1038/s41467-019-10059-6> |  |  |  |  |  |  |
|  |  |  |  |  |  |  |  |  |  |  |
|  |  |  |  |  |  |  |  |  |  |  |
|  |  |  |  |  |  |  |  |  |  |  |
|  |  |  | **Legend:** | Highest scoring (LO score) motif (known interaction mode & not buried) | In the top 5 motifs (known interaction mode & not buried) | In the top 15 motifs (known interaction mode & not buried) | In at least one fragment (all interaction types, buried and unburied) | Not found |  |  |
|  |  |  |  |  |  |  |  |  |  |  |

w/ str.: structure of interaction experimentally determined; w/o str.: structure not experimentally determined
